# Temporal dynamics in gastrointestinal helminth infections of sympatric mouse lemur species (*Microcebus murinus* and *Microcebus ravelobensis*) in Northwestern Madagascar

**DOI:** 10.1016/j.ijppaw.2024.100972

**Published:** 2024-08-05

**Authors:** Annette Klein, Ute Radespiel, Andrea Springer, Romule Rakotondravony, Christina Strube

**Affiliations:** a Institute of Zoology, University of Veterinary Medicine Hannover, Bünteweg 17, 30559, Hannover, Germany; b Institute for Parasitology, Centre for Infection Medicine, University of Veterinary Medicine Hannover, Bünteweg 17, 30559, Hannover, Germany; c Ecole Doctorale Ecosystèmes Naturels (EDEN), University of Mahajanga, 5 Rue Georges V - Immeuble KAKAL, Mahajanga Be, B.P. 652, Mahajanga, 401, Madagascar; d Faculté des Sciences, de Technologies et de l’Environnement, University of Mahajanga, Campus Universitaire Ambondrona, B.P. 652, Mahajanga, 401, Madagascar

**Keywords:** Golden-brown mouse lemur, Grey mouse lemur, Endoparasites, Nematodes, Cestodes, *Subulura*, Prevalence, Seasonal variation

## Abstract

Madagascar's lemur populations are declining in dwindling habitats due to anthropogenic expansion and changing climatic conditions. Gastrointestinal parasites can be important indicators to assess the health status of threatened species. However, parasites, hosts and the environment are connected in complex interactions. The present study aimed to disentangle the impact of seasonal and several host-specific factors (sex, species, age, reproductive status, and body mass) on endoparasitism in two small-bodied, co-occurring lemur species (*Microcebus murinus* and *Microcebus ravelobensis*) in the Ankarafantsika National Park. Helminth prevalence and egg shedding intensity was investigated via copromicroscopic examination of 810 fecal samples that were obtained from 178 individuals across an 11-month period with a longitudinal approach via repeated captures in a 30.6 ha forest area. Both mouse lemur hosts shed seven morphologically distinct egg types (assigned to *Subulura baeri*, unidentified Enterobiinae, *Spirura* sp., *Lemuricola* sp., two Hymenolepididae spp., one unidentified ascarid). Postmortem examination of two deceased individuals enabled assignment of adult worms to egg morphotypes of *S. baeri*, *Spirura* sp. and one Hymenolepididae sp., supported by molecular analysis. A significant seasonal variation was observed in the occurrence of the three most common helminth species *S*. *baeri* (total prevalence 71%), unidentified Enterobiinae (46%) and *Spirura* sp. (38%), with a higher likelihood of infection with advancing dry season. Neither host species, sex nor reproductive status had a significant effect on gastrointestinal helminth infections. Host body mass showed pronounced seasonal changes but did not differ significantly between infected and non-infected individuals. The pathogenic effects of gastrointestinal helminths therefore likely remained within compensable limits in the studied mouse lemur populations. Our findings highlight the prominent influence of seasonal changes on helminth communities. The results of combined morphologic and genetic approaches can furthermore help to overcome limitations of parasite identification via copromicroscopy by linking egg morphology to DNA sequences.

## Highlights

• Both studied mouse lemur species shed seven morphologically distinct egg types.
• Three egg types could be linked to adult morphology and generated DNA sequences.
• Prevalence of the most common helminth species increased with advancing dry season.
• No influence of intestinal helminth infection on host body mass was observed.

## Graphical abstract

**Figure:**
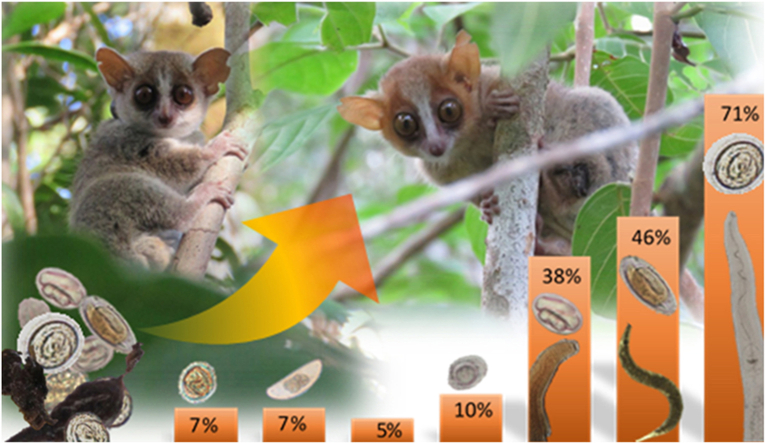

## 1 Introduction

Parasites account for a large part of global biodiversity and are ubiquitous in primates, with complex interrelations between host species, parasites and the environment in a given ecosystem. Although gastrointestinal parasites often cause little to no harm, they may become pathogenic in a host facing multiple simultaneous stressors either directly through pathological effects induced by the parasite (e.g. blood loss, tissue damage) or indirectly by reducing host condition (Chapman et al., 2006). Intestinal helminths in particular may interfere with digestion and nutrient absorption, leading to reduced energy uptake, reproductive success and survival (Coop and Kyriazakis, 1999; Blackwell et al., 2015; Akinyi et al., 2019), and may facilitate coinfections with other pathogens (Marcogliese and Pietrock, 2011). Investigation of parasite communities and potential factors driving patterns of parasitism in wild populations can hence be essential in the context of conservation biology.

One major challenge when investigating intestinal helminth infections in wildlife however, is our incomplete knowledge on the extant parasite diversity. It has been estimated that the number of parasitic helminth species exceeds the number of vertebrate hosts by at least 50% (Poulin and Morand, 2000; Dobson et al., 2008) and many host species have not even been investigated for their gastrointestinal parasites yet. Consequently, we can assume that only a fraction of the world's parasitic fauna is known to date. For example, for the 107 known Malagasy lemur host species, only 23 intestinal helminth species have been described to date (Irwin and Raharison, 2009; Vaughan, 2020). Studies on wildlife are often limited to a non-invasive approach for ethical reasons, using copromicroscopy for intestinal parasite identification due to its practicability and cost efficiency. The diagnostic power of morphological characterization of eggs and larval stages, however, is limited, and the complementary use of molecular sequencing techniques has indeed revealed a large number of morphologically similar cryptic parasitic taxa in recent years (Nadler and de León, 2011). Adult helminth specimens with higher informative morphological value are often unavailable and data on fully described parasite species in wildlife populations therefore remain scarce.

Host-parasite interactions are complex and several biotic and abiotic factors have been identified as being influential for infection dynamics in primates (Stuart and Strier, 1995; Nunn et al., 2006). The literature provides a heterogeneous picture of the effect of host dependent factors on intestinal parasites. An increase in population density, group size and sociality, for example, has been associated with higher intestinal parasite species richness and prevalence of infection in several primate species, such as Tana River red colobus (*Procolobus rufomitratus*), Tana crested mangabeys (*Cercocebus galeritus*) and brown spider monkeys (*Ateles hybridus*) (Vitone et al., 2004; Mbora and McPeek, 2009; Rimbach et al., 2015). Snaith et al. (2008), however, found parasite prevalence to be negatively related to group size and group spread in red colobus monkeys (*Procolobus rufomitratus*).

Some authors documented a significant influence of age and sex on parasitism in chacma baboons (*Papio ursinus*) (Benavides et al., 2012) and Japanese macaques (*Macaca fuscata yakui*) (MacIntosh et al., 2010). In chacma baboons, a convex relationship was observed between parasite species richness and host age, with young and old individuals harboring fewer intestinal parasite species. Individuals in better body condition furthermore exhibited lower parasite species richness. In Japanese macaques, a higher prevalence and fecal egg counts of directly transmitted nematodes in juveniles than adult conspecifics, as well as a male bias for those parameters may be suggestive of an immunological influence on parasitic infection pattern.

A male bias in parasitic infections has been found in numerous mammalian and bird species, with the immunosuppressive properties of sex steroids discussed as an underlying cause (Klein, 2004). Fecal testosterone and cortisol for example have been positively associated with helminth and protozoan parasite richness in male chimpanzees (Muehlenbein, 2006). A higher prevalence in intestinal helminth infection was furthermore found in male grey mouse lemurs (*Microcebus murinus*) and fat-tailed dwarf lemurs (*Cheirogaleus medius*) in western Madagascar (Springer and Kappeler 2016).

On the environmental scale, temperature and humidity have been proposed as influential on parasite infection patterns with higher prevalences and fecal egg counts under wet conditions (Stuart and Strier, 1995; Setchell et al., 2007; Raharivololona and Ganzhorn, 2010; Trejo-Macías and Estrada, 2012). However, none of these effects is universal, which emphasizes the complex interplay of various factors in a given ecosystem.

In the present study, we aimed to investigate the influence of several of these potentially predictive variables on intestinal helminth prevalence and egg shedding intensity in two closely related sympatric mouse lemur species. The grey mouse lemur (*Microcebus murinus*) and the golden-brown mouse lemur (*Microcebus ravelobensis*) are of comparable small body size with no sexual dimorphism and coexist in the same habitat in the strongly seasonal, dry deciduous forests of the Ankarafantsika National Park in northwestern Madagascar (Zimmermann et al., 1998). Both species are nocturnal, arboreal primates and promiscuous seasonal breeders with an onset of reproductive activity towards the end of the dry season (Radespiel, 2000; Randrianambinina et al., 2003). Their diet is omnivorous, consisting of plant matter (fruits, flowers, gum, leaves, buds and nectar), insect secretions and invertebrates as well as small vertebrate prey (Radespiel et al., 2006; Dammhahn and Kappeler, 2008; Thorén et al., 2011). Despite given similarities, some distinct differences have been recognized in their socio-ecology, in particular regarding daytime sleeping sites and the social grouping patterns during daytime resting: *M. ravelobensis* has been observed in mixed-sex sleeping groups in relatively open vegetation (Radespiel et al., 2003, 2009), while *M. murinus* uses tree holes as daytime sleeping sites. The degree of sociality furthermore varies between sexes in *M. murinus* with males sleeping solitarily, while females may be found in stable sleeping groups of related individuals (Radespiel et al., 1998, 2001).

We investigated patterns of intestinal parasite infections of the two mouse lemur species via copromicroscopy over two sampling periods totaling eleven months that covered the complete dry and partial rainy season. This longitudinal study design permitted integrative analyses of seasonal dynamics (environmentally driven and/or related to host reproduction) in addition to host related factors (species, age, sex, and body mass). We expected an increase in intestinal parasite prevalence under humid conditions as a consequence of favorable conditions for survival of parasite developmental stages in the environment. We furthermore predicted a positive influence of sociality (via more frequent or longer body contacts among co-sleepers) on parasite species with a direct life cycle. A certain degree of acquired immunity may counteract gastrointestinal parasite infection in older individuals, and we therefore expected higher prevalence and parasite species richness in juveniles. The immunosuppressive properties of testosterone may furthermore lead to a male bias in parasitism in mature hosts. Host body mass reflects the individuals' energy metabolism and we predicted a lower body mass in individuals with a higher gastrointestinal parasite intensity as a result of the parasites’ interference with host nutrition.

The recovery of two dead mouse lemurs from the forest and subsequent necropsy in the present study furthermore provided the unique opportunity to match egg and adult helminth morphology with molecular results. We thus aimed for a more precise taxonomic classification of the respective intestinal helminth species.

## 2 Material and methods

### 2.1 Study site and mouse lemur trapping

The protected forest area of the Ankarafantsika National Park covers about 135,000 ha in the Boeny Region of northwestern Madagascar and is subject to a pronounced seasonality with a hot and humid rainy season from November to April and a relatively cooler dry season from May to October. This study was conducted between April and November 2015 and from March to May 2016 in the Jardin Botanique A (JBA, 16°19′S, 46°48′E), a 30.6 ha rectangular area of dry deciduous forest that is accessible by a system of trails. Climatic conditions and trapping procedures are detailed by Klein et al. (2018). Briefly, free ranging *M. murinus* and *M. ravelobensis* were trapped using Sherman life traps six nights per month, measured, weighed, and sexed, and marked with a small subcutaneous transponder (Trovan ID-100; Telinject®, Römerberg, Germany) for lifelong identification upon first capture. Host species were identified based on morphologic characteristics (Zimmermann et al., 1998). Female reproductive status was classified as “inactive” (no morphological changes), “cyclic” (reddening, swelling, or opening of vulva), “pregnant” (weight gain with fetuses perceptible during abdominal palpation) or “lactating” (morphological changes in mammary glands, milk secretion). Reproductive stage of males was divided into “testis small” (no testes palpable) and “testis large” (visible scrotum with palpable testicles). Age classes (adult, juvenile) were determined by a combination of body mass, morphology, and presence/absence of reproductive changes.

### 2.2 Fecal sampling and copromicroscopic analyses

Fecal samples were collected either directly if the individual defecated during handling, or from the trap. Traps were washed with commercial soap after the individual was released and disinfected with 90% ethanol prior to re-installment in the forest. Samples were stored in 90% ethanol in 2 ml tubes and kept refrigerated until further processing. If an individual contributed more than one fecal sample within a three-night trapping interval, only one sample (preferably a fresh sample collected during handling or alternatively the one with the largest sample volume) was used for copromicroscopic examination. Samples were centrifuged at 1400×*g* for 5 min to remove the ethanol supernatant from the fecal pellet. The fecal mass was determined, then 1.5 ml of saturated zinc sulfate solution (specific gravity: 1.3) was added to the feces and thoroughly mixed with a spatula. Flotation of parasite stages was enhanced by centrifugation at 250×*g* for 10 min. The supernatant was then washed through a sieve with tap water into a 15 ml tube and centrifuged at 450×*g* for 5 min to sediment the parasite stages. Supernatant water was discarded and the sediment transferred into a counting chamber. Microscopic identification of parasite stages was in accordance with Kiene et al. (2021). A complete count of the sediment was conducted to determine egg shedding intensity in terms of number of eggs per gram feces [EPG].

### 2.3 Post-mortem examination and identification of adult helminths

In October 2015, one male adult *M. ravelobensis* was found dead in the forest. Post-mortem examination revealed hemorrhages in the dorsal and lateral right neck area as well as blood-filled lungs that may have resulted from a potential predator attack. Five fecal samples had been collected from this individual previously. A female *M. murinus* was found dead in April 2016, and a fractured skull suggested trauma to the head as the most likely cause of death in this individual. Thirteen fecal samples were available for this animal.

Upon post-mortem examination, the gastrointestinal tract was opened and ingesta samples as well as macroscopically visible helminths were stored in 90% ethanol. A sample of the collected specimens (N = 14) was embedded in Berlese's mounting medium for morphological identification. Helminth specimens were examined under light microscopy (Axiophote microscope, Carl Zeiss MicroImaging, Jena, Germany), photographed with a Colorview IIIu Camera, and measured using cell^B Image Acquisition Software (version 3.1; Olympus Soft Imaging Solutions, Hamburg, Germany). Eggs from mature female specimens were used for morphologic examination. Parasite morphology was compared to available descriptions of nematodes and cestodes available for Malagasy lemurs (Chabaud et al., 1965; Hugot et al., 1995, 1996; Irwin and Raharison, 2009; Raharivololona, 2009; Clough, 2010; Robles et al., 2010; Hugot and Robles, 2011).

### 2.4 Molecular analysis

Samples for molecular analysis were selected based on morphological resemblance under light microscopy to cover the different helminth morphotypes recovered from both mouse lemur species during post-mortem examination. DNA from four adult specimens was isolated using DirectPCR® Lysis Reagent (Cell) (PeqLab, Erlangen, Germany) according to the manufacturers’ instructions. The ITS1–5.8S–ITS2 rDNA region was successfully amplified for two nematodes and one cestode extracted from the intestines using primers NC2 and NC5 (Newton et al., 1998) following the protocol by Klein et al. (2019). This region could not be successfully amplified for a nematode recovered from the stomach of the *M. ravelobensis*. Therefore, the cytochrome oxidase subunit I gene (COI) of this specimen was targeted using primers LCO1490 and HCO2198 (Folmer et al., 1994) according to Klein et al. (2019). PCR products were Sanger-sequenced at Microsynth Seqlab Sequence Laboratories (Göttingen, Germany). Obtained nucleotide sequences were compared to publicly available sequences via BLAST search and aligned using Geneious v10.2.6 (Biomatters Ltd., Auckland, New Zealand). Generated sequences were deposited in NCBI GenBank under accession numbers **PP726880** and **PP734023-PP734025**.

### 2.5 Statistical analyses

Total prevalences were calculated for seven morphologically distinct egg types for both studied mouse lemur species. An individual was considered positive if at least one fecal sample contained the respective egg type over the course of the whole study period. Parasite species richness was assessed as the number of different parasite taxa detected per sample.

Taxon-specific presence/absence data was analyzed in more detail by fitting generalized linear mixed effect models (GLMMs) with binomial error structure and logit-link function on the basis of all analyzed samples. We tested the following fixed effects (predictive variables): host species (*M. murinus, M. ravelobensis*), sex, age (juvenile, adult), reproductive status (females: inactive, cyclic, pregnant, lactating; males: testis large, testis small), sample mass, season (rainy season, dry season), and the temporal factor sampling month. A potential effect of presence/absence of the six respective other egg morphotypes was also tested. A comparison of the two periods sampled in both years, April–May 2015 and April–May 2016, revealed no differences between study years for the investigated egg morphotypes and data was therefore pooled across both years. Sampling month was included as an interaction-term for reproductive status to account for the fact that both studied mouse lemur species are seasonal breeders. To account for repeated sampling of individuals, animal identity was included as a random-effect term in all models. Potential predictive variables were tested individually and successively added to the model if influences were significant and/or improved the final model. Models, including a null-model with no predictive variables, containing only the random factor, were compared via likelihood ratio tests and model fit was evaluated based on the Akaike information criterion (AIC).

A potential influence of the predictive variables host species, sex, age, season, and sampling month on egg shedding intensity (number of eggs per gram feces [EPG], log-transformed), was tested by including all positive samples for the respective egg morphotypes in linear mixed effects models (LMEs). Residuals were calculated for all LMEs and evaluated for normal distribution and homogeneity.

A potential link between parasite occurrence and host body mass was analyzed in the subset of adult individuals only (N = 154). Missing body mass data led to a dropout of 29 samples (4.4%) in this dataset. Individual body mass was modelled as a response variable in a linear mixed effects model with the predictive factors species, sex, month and presence/absence of the respective egg morphotype.

Significant predictor variables with multiple factorial groups were subjected to post-hoc analyses, computing all pairwise differences (in analogy to Tukey's test) based on the parameters of the fitted model. Statistics were performed in R version 3.5.2 (R Core Team, 2018) using packages lme4 (Bates et al., 2014) and multcomp (Hothorn et al., 2008).

## 3 Results

### 3.1 Endoparasite eggs and (oo-)cysts detected in fecal samples

A total of 810 fecal samples from 178 individuals (79 *M. murinus* [37 females, 42 males] and 99 *M. ravelobensis* [54 females, 45 males]) were examined copromicroscopically (Table 1). The number of samples analyzed per individual ranged from one to nineteen with a mean of 4.5 samples over the course of the study period (Additional file Table 1).

**Table 1:** Monthly and seasonal sample distribution (T = total, M = male, F = female).

	MAR 2016 (N = 29)			APR 2015/16 (N = 69)			MAY 2015/16 (N = 157)			JUN 2015 (N = 96)			JUL 2015 (N = 113)			AUG 2015 (N = 90)			SEP 2015 (N = 115)			OCT 2015 (N = 101)			NOV 2015 (N = 40)			Rainy season	Dry season
	T	M	F	T	M	F	T	M	F	T	M	F	T	M	F	T	M	F	T	M	F	T	M	F	T	M	F	T	T
*M. murinus*	**15**	2	13	**33**	13	20	**69**	34	35	**39**	14	25	**39**	20	19	**35**	19	16	**54**	28	26	**44**	24	20	**16**	7	9	**64**	**280**
*M. ravelobensis*	**14**	4	10	**36**	15	21	**88**	31	57	**57**	21	36	**74**	29	45	**55**	25	30	**61**	32	29	**57**	33	24	**24**	14	10	**74**	**392**

Rainy season: November–April; Dry season: May–October.

**Fig. 1:**
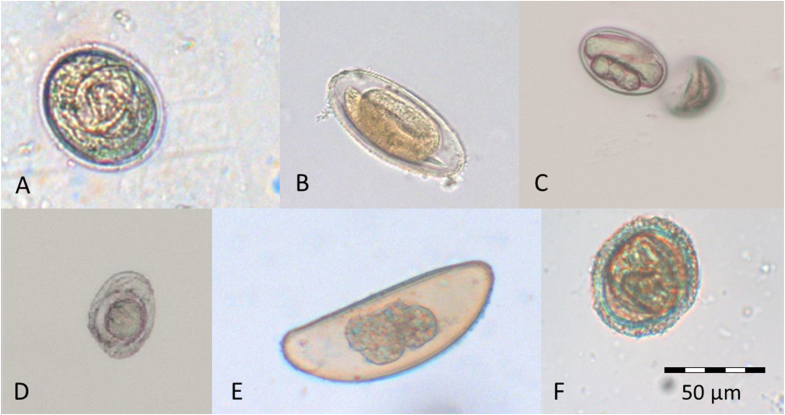
Egg morphotypes detected in fecal samples of *M. murinus* and *M. ravelobensis*: (A) *Subulura baeri*, (B) unidentified Enterobiinae, (C) *Spirura* sp., (D) *Hymenolepis* sp. 1, (E) *Lemuricola* sp. and (F) ascarid egg.

Seven morphologically distinct egg morphotypes as well as adults of one of the detected egg morphotypes were identified in the samples. These egg morphotypes (5 nematodes, 2 cestodes; Fig. 1) occurred with largely different total prevalences (Table 2). *Subulura baeri* eggs were the most frequently detected intestinal parasite stages with a total prevalence of 71.3%. Eggs of unidentified Enterobiinae and *Spirura* sp. were excreted by 46.6 % and 38.2% of individuals, respectively, at least once over the course of the whole study period. Egg morphotypes resembling *Lemuricola* sp. were identified in at least one fecal sample of 7.3% of examined individuals, and eggs of an unidentified ascarid were detected with a total prevalence of 7.3%. Two cestode egg types were detected, *Hymenolepis* sp. 1 and *Hymenolepis* sp. 2 (Raharivololona, 2009), with a total prevalence of 10.1% and 5.1%, respectively.

**Table 2:** Total prevalence of gastrointestinal parasites detected in fecal samples of the mouse lemur species *M. murinus* and *M. ravelobensis* in the Ankarafantsika National Park during 2015–2016. Note that an individual was considered positive if at least one fecal sample contained the respective egg type over the course of the study period.

Parasite morphotype	Overall (N = 178)	*M. murinus* (N = 79)	*M. ravelobensis* (N = 99)
*Subulura baeri*	71.3%	69.6%	72.7%
Unidentified Enterobiinae	46.6%	35.4%	55.6%
*Spirura* sp.	38.2%	38.0%	38.4%
*Lemuricola* sp.	7.3%	7.6%	7.1%
Ascarid eggs	7.3%	7.6%	7.1%
*Hymenolepis* sp. 1	10.1%	11.4%	9.1%
*Hymenolepis* sp. 2	5.1%	7.6%	3.0%

Coccidian-like structures were occasionally observed but could not be extracted successfully from the sedimentation solution for sufficient microscopic identification under higher magnification. The same was the case for cyst-like structures of potential *Entamoeba* species.

### 3.2 Adult helminths identified in fecal samples

**Fig. 2:**
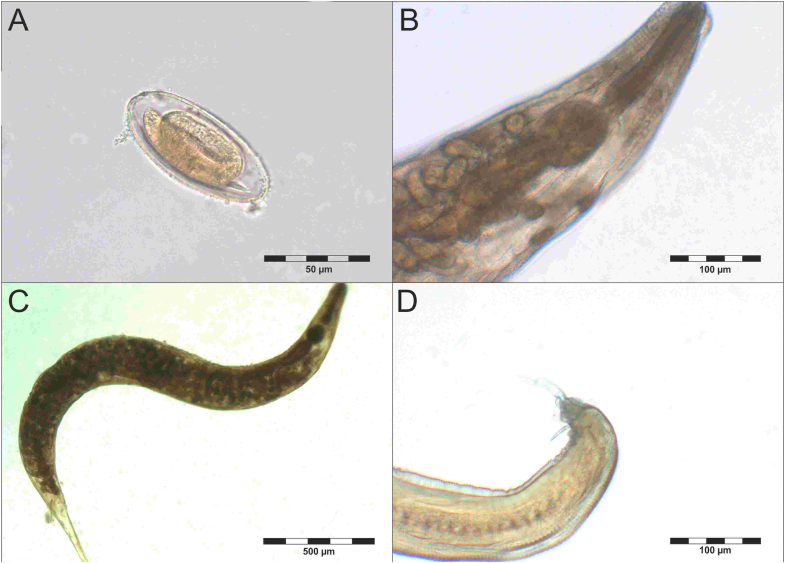
Unidentified Enterobiinae extracted from fecal samples of *M. murinus* and *M. ravelobensis*: (A) egg (B) anterior end (C) adult female specimen (D) posterior end of an adult male specimen.

Adult helminths found in mouse lemur feces were morphologically classified as unidentified Enterobiinae (Fig. 2). They were present in 13.7% of fecal samples that also contained the respective egg morphotype (N = 153), whereas no sample contained adult Enterobiinae only. Both sexes possessed a cuticle with transverse striations, lateral alae at the apical extremity, and a club-shaped esophagus with a short isthmus and a round esophageal bulb (Fig. 2B). Female specimens measured 1775 μm to 2453 μm (N = 4) with the vulva located midway of body length and the posterior end tapering into a pointed tail (Fig. 2C). Male specimen measured 866 μm and 998 μm and had a blunt posterior end with one spicule (Fig. 2D). Eggs of unidentified Enterobiinae were elongated ovals with 79.57 μm in length (71.97–82.65 μm N = 15) and 34.39 μm in width (32.03–38.76 μm N = 15) containing a coiled embryo (Fig. 2A).

### 3.3 Adult helminths identified by post-mortem examination and molecular analysis

Adults of three helminth species were obtained during post-mortem examination and eggs of mature females could be morphologically matched to the corresponding eggs in fecal samples.

**Fig. 3:**
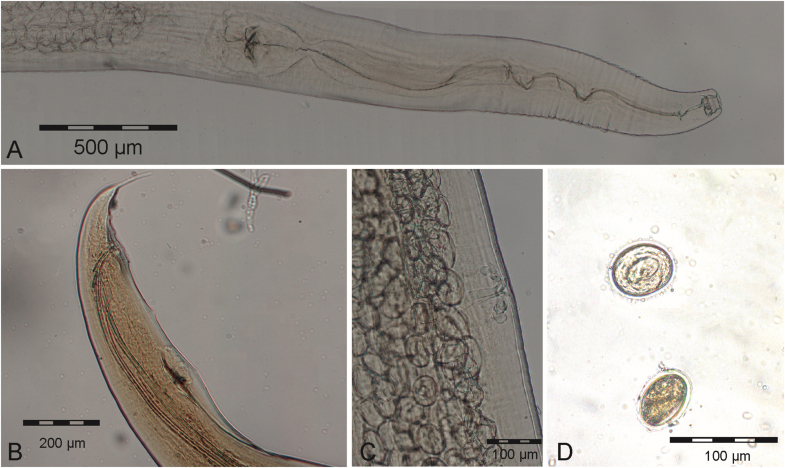
*Subulura baeri* from *Microcebus* hosts: (A) anterior end of an adult female specimen, (B) posterior end of an adult male specimen, (C) vulva, and (D) eggs with characteristically coiled embryo (top) and lateral view (bottom).

Both dissected mouse lemurs harbored male and female nematodes morphologically consistent with *Subulura baeri* (Nematoda: Subuluridae) previously described by Chabaud et al. (1965) based on specimens obtained from *M. murinus* at the same study site. Specimens were present throughout the small and large intestines but occurred predominantly in large numbers in the caecum. Forty-eight specimens (20 females, 28 males) were recovered from the caecum of the dissected *M. murinus*, three from the small intestine (two females, one male) and ten (seven females, three males) from the large intestine. From *M. ravelobensis*, 64 specimens were extracted, all from the caecum (35 females, 29 males). The embedded female specimens (N = 7) ranged from 11,458 to 17,947 μm in length, embedded male specimens (N = 3) from 8024 to 9296 μm. The cuticle was striated and tapered to both extremities. The anterior end was blunt with a buccal cavity approximately as long as wide and a rhabditiform esophagus. Cervical alae were absent in both sexes (Fig. 3A). In males, the posterior body end was bent ventrally. The spicules were of equal length, measuring 1154 to 1347 μm. Caudal papillae were arranged in three precloacal and six postcloacal pairs, with the first pair situated lateral to the anterior margin of an oval precloacal sucker (Fig. 3B). In females, the vulva (Fig. 3C) was situated slightly anterior to mid-body, while the tail was about 5 % of the body length. Eggs were ovoid to round, measuring 57 μm in diameter and contained a coiled embryo (Fig. 3D).

Sequences of the ITS1–5.8S–ITS2 region amplified from specimens recovered from both dissected individuals showed more than 99% pairwise identity (GenBank acc. no. **PP734024** and **PP734025**). They furthermore aligned with only 1 and 4 ambiguous sites (99.90% and 99.62% pairwise identity, query cover 100%), respectively, with a sequence generated from eggs extracted from feces of a *M. ravelobensis* (acc. no. MW520852) by Kiene et al. (2021). A further comparison of the generated sequences with publicly available data via BLAST search identified 82% nucleotide identity with *Subulura chinensis* (acc. no. MK770149, query cover: 72% and 77%). A sub-analysis of the 5.8S rRNA region, which only allows accurate classification on a higher taxonomic level (Chilton, 2004), revealed a pairwise identity of >99% with a published sequence of *S*. *chinensis*, confirming correct genus identification. No sequence of *S. baeri* was found in public databases.

**Fig. 4:**
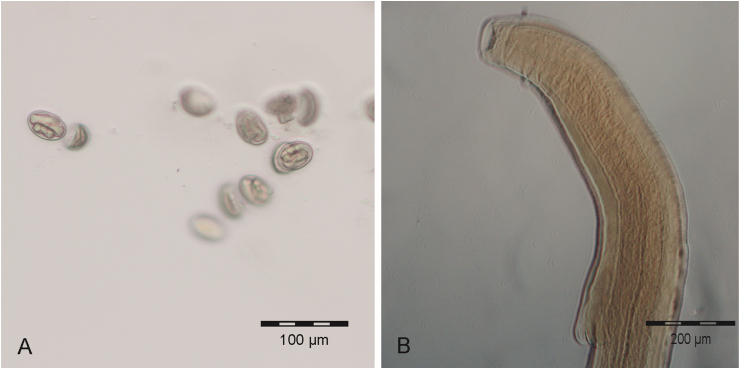
*Spirura* sp. recovered from *M. ravelobensis* during post-mortem examination: (A) eggs and (B) anterior end with doubled cuticular ventral boss.

Moreover, elongated, reddish nematodes (twelve males and one female) were recovered from the stomach of the dissected male *M. ravelobensis*. Their morphology showed similarities to the original description of *Spirura diplocyphos* (Nematoda: Spiruridae) (Chabaud et al., 1965) based on immature specimens detected in the stomach of a *Cheirogaleus medius* in the Ankarafantsika National Park. The mature female worm of the present study measured 26,225 μm in length, broadening towards the posterior end. Embedded males were distinctly smaller, with only 12,705 μm to 13,427 μm in length (N = 2). The esophagus was long (approximately 35 % of body length) with indistinct demarcation between the muscular and glandular part. Both sexes possessed a conspicuous doubled cuticular ventral boss (Fig. 4B). Eggs extracted from the female specimen were ovoid and measured on average 46 μm in length (38 μm–52 μm, N = 30) and 29 μm in width (25 μm–34 μm, N = 30) (Fig. 4A). A 602 bp fragment of the COI gene (GenBank acc. no. **PP726880**) showed highest consensus with *Protospirura muricola* (acc. no. KP760207) in BLAST search, with 83.8% pairwise identity (query cover: 100%).

**Fig. 5:**
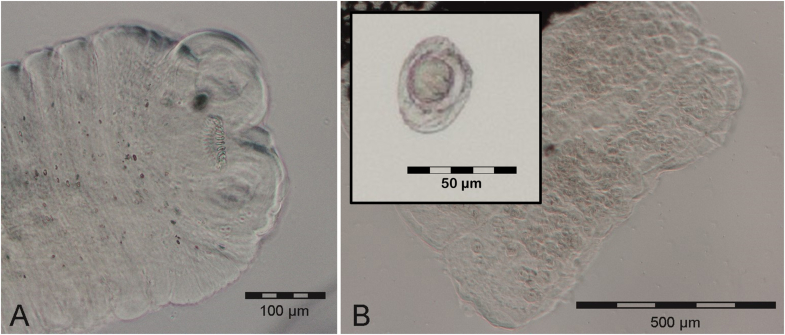
Hymenolepididae recovered from the small intestine of a female *M. murinus*: (A) scolex, (B) gravide proglottids and eggs (insert).

Three cestodes were removed from the small intestines of the female *M. murinus.* The scolex of one embedded specimen measured 200 μm in length and 335 μm in width and contained four suckers and a retractable rostellum, armed with 24 hooks (Fig. 5A). A neck region was not identifiable by light microscopy. All three specimens were incomplete at the posterior end but had a clearly segmented strobila of >100 proglottids, all of which were broader than long and increasing in width with progressing development. Immature proglottids of the embedded specimen exceeded the scolex in width, measuring 454 x 64 μm. Mature proglottids measured 916 × 68 μm, gravid proglottids 1159 × 183 μm (Fig. 5B). The pale eggs were ovoid to subspherical, 43 × 36 μm in size, containing the oncosphere with two to three pairs of embryonic hooks (Fig. 5B insert). This egg morphotype was detected in the feces of 10.1% of mouse lemurs. The obtained specimens showed morphological resemblance to *Hymenolepis* sp. 1, described by Raharivololona (2009). Comparison of a 914 bp rDNA fragment containing the partial ITS1, full 5.8S and partial ITS2 region (GenBank acc. no. **PP734023**) with publicly available data via BLAST showed the highest pairwise identity with *Staphylocystis schilleri* (acc. no. KF257896, pairwise identity: 88.35%, query cover: 99%) and *Hymenolepis microstoma* (acc. no. LR215990, pairwise identity: 88.44%, query cover: 95%). The analysis on the 5.8S region suggested the classification into the family Hymenolepididae.

### 3.4 Influence of host and environmental factors on parasite occurrence and egg shedding intensity

Only parasite species with a total prevalence >15% (*Subulura baeri*, unidentified Enterobiinae and *Spirura* sp.) were submitted to full statistical modelling of all potential predictive variables, since the highly irregular distribution of the rarer egg types precluded the calculation of reliable models for variables with multiple factorial groups. However, none of the binomial factors host species, age or sex had a significant influence on the detection of *Hymenolepis* sp. 1, *Hymenoepis* sp. 2, *Lemuricola* sp. or ascarid egg morphotypes, with no model explaining the respective dataset better than the null-model. Furthermore, fecal mass had no significant effect on the likelihood of egg detection for any of the seven egg morphotype.

**Fig. 6:**
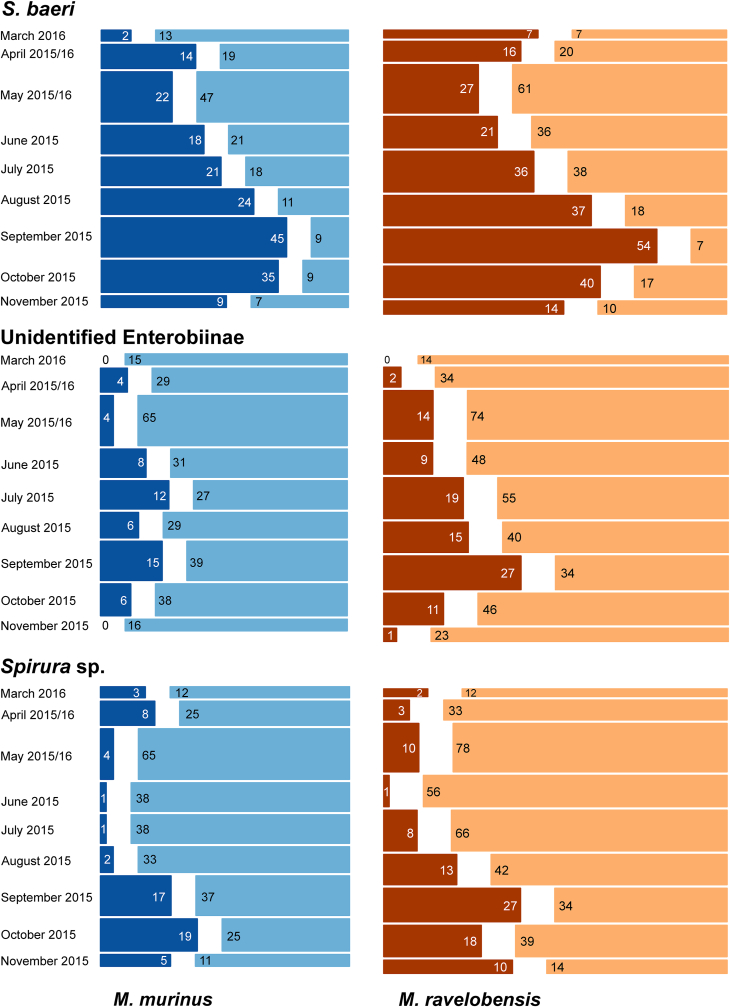
Monthly frequency of *S. baeri*, unidentified Enterobiinae and *Spirura* sp. in all examined fecal samples of *M. murinus* and *M. ravelobensis*. Positive samples are shown in dark blue for *M. murinus* and dark brown for *M. ravelobensis*, negative samples are in light blue for *M. murinus* and light brown for *M. ravelobensis*. Bar widths indicate sample size for the sampling month(s) and numbers indicate respective positive/negative samples. (For interpretation of the references to colour in this figure legend, the reader is referred to the Web version of this article.)

The multivariate modelling for the three most frequent egg types (*Subulura baeri*, unidentified Enterobiinae and *Spirura* sp.) showed that they were not excreted homogenously across months, but occurrence increased over the course of the dry season with a peak in September for *S. baeri* and unidentified Enterobiinae and a higher level plateau for *Spirura* sp. from September to November (Fig. 6, for details see Additional file Table 2). This seasonal pattern was consequently also reflected by an increase in parasite species richness. No significant influence of host species, host sex, or host reproductive status was detected for any of the three egg morphotypes. These parameters did not help to predict the observed variation in intestinal parasite occurrence and were therefore not included in the final statistical model. However, age was identified as a significant factor for *S. baeri*, with a higher likelihood of infection in adults compared to juvenile individuals (GLMM: Estimate = −0.878, *P* < 0.001). No age effect was observed for the other two species. An infection with *S. baeri* had a positive effect on the likelihood of infection with unidentified Enterobiinae (GLMM: Estimate = 0.840, *P* < 0.001) and *Spirura* sp. (GLMM: Estimate = 1.537, p < 0.001). *Subulura baeri* occurrence in turn was positively influenced by unidentified Enterobiinae (GLMM: Estimate = 0.884, *P* < 0.001) and *Spirura* sp. (GLMM: Estimate = 1.385, *P* < 0.001), while occurrence of *Lemuricola* sp. was associated with a lower likelihood of *S. baeri* infection (GLMM: Estimate = −1.845, *P* = 0.014).

**Fig. 7:**
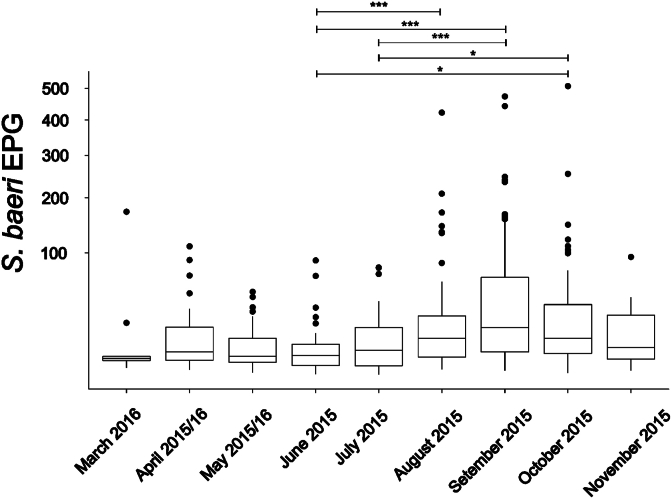
*S. baeri* egg shedding intensity (eggs ger gram feces [EPG]) across the different sampling months.

The number of eggs per gram feces was not normally distributed for any of the three nematode species investigated in more detail. Egg shedding intensity varied between 0.71 and 507.69 EPG for *S. baeri* (N = 442), 1.04 and 95.24 EPG for unidentified Enterobiinae (N = 145), and 0.72 and 222.22 EPG for *Spirura* sp. (N = 151). The temporal pattern observed in *S. baeri* occurrence was also reflected in the EPG counts of this species (Fig. 7, Additional file Table 3). No other factor had a significant influence on *S. baeri* egg shedding intensity. A significant effect of season was identified for unidentified Enterobiinae with higher egg counts in the rainy than in the dry season (LME: Estimate = −0.435, *P* = 0.023). None of the tested variables had a significant effect on egg shedding intensity in *Spirura* sp., with no factor explaining variation better than the null model.

None of the parasite species had a significant effect on host body mass (Additional file Table 4). Statistically significant variations in body mass of adult mouse lemurs were solely attributed to species-specific differences and sampling month as previously shown during the investigation of ectoparasite infestation in the same study population (Klein et al., 2018).

## 4 Discussion

### 4.1 Gastrointestinal helminth diversity

In the present study, seven morphologically distinct egg types were detected via copromicroscopy in *M. murinus* and *M. ravelobensis*. Microscopic examination of fecal samples is a common approach to evaluate gastrointestinal parasite infections due to its practicability, cost efficiency and the validity of positive results when performed by an experienced investigator. However, species designation of detected egg morphotypes based on morphologic characteristics is limited (Chilton, 2004). Eggs of closely related species often show only subtle to no discernible differences, and only a fraction of the species parasitizing wildlife is fully described (Gillespie, 2006; MacPhee and Greenwood, 2013). A comprehensive description of new species requires a representative number of adult male and female specimens obtained during post-mortem examination or passed in feces, e.g., following anthelmintic treatment. In the field of wildlife medicine, these samples are often unavailable due to practical and/or ethical reasons. With the advancement of molecular techniques, DNA-based methods have gained importance in diagnosis of intestinal helminth infections and amplification and sequencing of targeted DNA fragments from eggs, larvae or adult worms is now widely used for species identification (McManus and Bowles, 1996). However, equally to the traditional morphological approach, the diversity of gastrointestinal parasite species infecting wildlife based on genetic data has only been explored to a limited extent.

Most species descriptions of endoparasites of Malagasy lemurs date back to the mid-20th century, driven by the works of Alain Chabaud, Eduard Brygoo and Annie Petter. More recent studies using non-invasive approaches and assessment of gastrointestinal parasite burden and species diversity are based on microscopic and/or genetic investigation of fecal matter. The focus furthermore shifted from a fundamental parasitological perspective to multidisciplinary studies including parasites as just one aspect in the context of, for example, health assessments or ecological questions (Irwin and Raharison, 2009). Available detailed morphological descriptions of Malagasy gastrointestinal parasites are therefore limited. To our knowledge, there is no study to date providing adult morphological characteristics and corresponding molecular data.

In our study, the death of two mouse lemurs during the period of field work enabled extraction of adult helminths from the gastrointestinal tract during post-mortem examination and thus allowed assignment of egg morphotypes to adult specimens and thereby identification of eggs encountered during copromicroscopy on a lower taxonomic level.

#### 4.1.1 Subulura baeri

Our morphological and genetic data show that both mouse lemur species are parasitized by *S. baeri*, with the most frequently detected egg type during microscopic screening of fecal samples assigned to this species. Chabaud et al. (1965) reported *S. baeri* from yet another member of the Cheirogaleidae family, the fat-tailed dwarf lemur (*Cheirogaleus medius*) at the same study site. The life cycle of this nematode has not been described yet, but an indirect life cycle with an intermediate insect host can be assumed given existing knowledge on other *Subulura* species (Abdou and Selim, 1963), which is in line with the omnivorous diet of *M*. *murinus*, *M. ravelobensis* and *C. medius*. Based on the post-mortem findings, the caecum appears as a predilection site for adult worms. Interestingly, Kiene et al. (2021) also isolated eggs of the Subuluroidea fam. gen. spp. morphotype from feces of the frugivorous Dormouse tufted-tailed rat (*Eliurus myoxinus*) as well as from *Rattus* *rattus* in the Ankarafantsika National Park. Molecular analyses, however, indicated two distinct parasite species of the same family in these two hosts. Egg morphotypes addressed as *Subulura* sp. have been documented in *Microcebus* and *Cheirogaleus* species from various areas (Raharivololona and Ganzhorn, 2010; Springer and Kappeler, 2016), as well as from Coquerel's giant mouse lemurs (*Mirza coquereli*) (Springer and Kappeler, 2016), red-fronted lemurs (*Eulemur rufifrons*) (Peckre et al., 2018) and ring-tailed lemurs *(Lemur catta*) (Loudon and Sauther, 2013). Whether they all in fact harbored the same nematode species remains to be investigated. The present study provides a basis for such studies by linking newly generated molecular data to the original detailed species description of *S. baeri* and thereby facilitating the comparison of egg types with little morphological diagnostic characteristics to a specific species using molecular tools.

#### 4.1.2 Spirura species

Another rather frequent gastrointestinal parasite egg type (overall prevalence = 38.2%) could be matched to an adult specimen of the genus *Spirura* from the stomach of *M. ravelobensis*. Unfortunately, no molecular data is available for any species of this genus in public databases. Only one species, *Spirura diplocyphos* has so far been described from Malagasy lemurs, based on an immature female from a *C. medius* sampled in the Ankarafantsika National Park (Chabaud et al., 1965). Given that the morphology of the gravid female parasite detected in the present study was similar to this previous species description, particularly by its conspicuous doubled cuticular ventral bossing, and that the original authors reported *S*. *diplocyphos* infections in *M. murinus* in the same study area, the parasite we examined might very well have belonged to this species, although the stage difference precluded a final decision on its taxonomic classification. The life cycle of spirurid nematodes includes an intermediate insect host. An overlap in used habitat and food resources has been discussed as the interface for parasite crossover between different insectivorous-omnivorous mammalian species (Torres et al., 2015) and the diets of both investigated mouse lemur species, *M. murinus* and *M. ravelobensis,* include invertebrate prey (Radespiel et al., 2006). It could therefore be expected that they share the same heteroxenous intestinal parasite species. Surprisingly, records of spirurid nematodes infecting terrestrial vertebrates in Madagascar are limited to the early work by Chabaud and colleagues (Chabaud et al., 1965) and a recent investigation on small mammals including mouse lemurs by Kiene et al. (2021). However, they were frequently found in a study on the intestinal parasite fauna of Malagasy birds (Vassiliades, 1970). Elsewhere, infections with nematodes of the genus *Spirura* have been described primarily in primates (Solórzano-García and de León, 2018), but also marsupials (Torres et al., 2015), tree shrews (Quentin and Krishnasamy, 1975), rodents (da Costa Cordeiro et al., 2015) and bats (Peralta-Rodríguez et al., 2012). Host species of morphologically defined *Spirura* species can span across distant taxonomic groups in some cases. Patent infections with *Spirura guianensis,* for example, have been documented in primates (*Saguinus nigricollis, Saguinus geoffroyi* (Solórzano-García and de León, 2018)*, Saimiri sciureus* (Blanchard and Eberhard, 1986)) as well as in marsupials (*Gracilinanus agilis* (Torres et al., 2015)*, Caluromys philander* (Quentin, 1973), *Philander opossum* (Amato et al., 1976)).

#### 4.1.3 *Hymenolepis* species

Cestodes from the small intestine of the female *M. murinus* were genetically assigned to the family Hymenolepididae and showed some similarities in egg and adult morphology to one of two unidentified species of the genus *Hymenolepis* described by Raharivololona (2009). The author addressed this tapeworm as *Hymenolepis* sp. 1. However, rostellar hooks were not identified in that species and no molecular data was generated in the respective study. The taxonomy of Hymenolepididae is under constant revision due to an increasing species diversity and molecular reassessments of conventional classifications (Sharma et al., 2016), containing numerous genera with hundreds of species (Neov et al., 2021). We therefore conservatively address the recovered cestode species only on the family level Hymenolepididae. Most cestodes of this family require an intermediate arthropod host to complete their lifecycle, however the zoonotic *Hymenolepis nana* is known for direct transmission (Ito and Budke, 2021). Hymenolepididae have been previously identified as parasites of Cheirogaleidae (Raharivololona, 2009; Hämäläinen et al., 2015c; Rafalinirina et al., 2015; Springer and Kappeler, 2016), rodents (Haukisalmi et al., 2010) and humans in Madagascar (Barrett et al., 2013), and their distribution range is predicted to increase. In 2013, Barrett et al. (2013) published modeling results for the current and future geographic distribution of lemur parasites using species distribution models and predicted an expansion over 60% in the potential distributions for *Hymenolepis* species. Species diversity, host specificity and zoonotic potential, and thus epidemiological significance of present *Hymenolepis* species require further investigation. The zoonotic properties of *Hymenolepis nana*, for example, complicate control strategies of this neglected tropical disease (Thompson, 2015).

### 4.2 Sources of variation in gastrointestinal helminth occurrence and egg shedding intensity

Host-parasite interactions have been subject of numerous studies and several factors can shape intestinal parasite infections in non-human primates. In the present study, we evaluated the influence of the host-specific factors species, age, sex, reproductive status and host body mass, as well as the environmental factors season and month. The low prevalences of both cestode egg morphotypes as well as *Lemuricola* sp. and the unidentified ascarid eggs precluded complex modelling for these parasite taxa. The low detection rates in the case of Hymenolepididae, however, are probably at least partly the result of a low sensitivity of copromicroscopy for tapeworm diagnosis. Cestode proglottids or eggs are released intermittently and are not distributed uniformly in feces, resulting in a high likelihood of false negative results and therefore underestimation of true prevalence (Mwape and Gabriël, 2014). In support of this interpretation, none of the thirteen fecal samples of the female *M. murinus* contained cestode eggs ante mortem, with the last sample taken two days prior to her death, even though three mature cestodes were recovered during necropsy. It is furthermore noteworthy that most statistically significant effects were inferred for *S. baeri*, the most frequently detected intestinal parasite species. These findings strongly suggest that large datasets and relatively high prevalences are needed to reliably disentangle seasonal and host-specific effects in complex host-parasite interactions.

#### 4.2.1 Seasonal variation

Multivariate modeling was possible for two heteroxenous parasites (*S. baeri, Spirura* sp.) and one directly transmitted, homoxenous parasite (unidientified Enterobiinae). For all of these the most important factor governing the likelihood of infections was the temporal factor sampling month. However, contrary to our expectation, occurrence were significantly higher in the late dry than in the early dry season or in the humid rainy season. Dynamics of parasitic infections are shaped by parasite development and survival as well as encounter frequency with infective parasitic stages. High temperatures and humidity in the rainy season as well as increased moisture levels in the forest soil due to high precipitation rates should positively influence parasite survival of all free-living stages (Stromberg, 1997). However, a higher availability of high-quality food resources, such as fruits and nectar in the higher forest strata may reduce the need of mouse lemur hosts to descend to the forest floor while foraging and may reduce exposure to infective parasitic stages. Indeed, a higher proportion of fruits, leaves and nectar was documented between October and April in the diet of *M. murinus* and *M. ravelobensis* (Thorén et al., 2011). A previous investigation on ectoparasites of the studied mouse lemur population also revealed a lower risk of tick infestation in the rainy season which was also attributed to a less frequent descend to the lower forest strata (Klein et al., 2018).

The dry season furthermore constitutes a demanding period for mouse lemurs with low food availability, high daily temperature fluctuation between cold nights and hot days and the social stress of the reproductive season. Hämäläinen et al. (2015a) found a seasonal increase in fecal glucocorticoid metabolites in *M. murinus* in the dry deciduous forest in central western Madagascar, indicative of a stress response to the energetically demanding conditions of the dry season. Feeding and nutrition furthermore have a complex interaction with immune status and parasitic infection. Under experimental conditions, it was shown that food limitation constrains the innate immune response against intestinal nematode infections in field voles (*Microtus agrestis*) (Forbes et al., 2016). We therefore assume that the observed seasonal pattern in gastrointestinal helminth infections is predominantly modulated by host-associated rather than environmental factors. The September peak in infection with the directly transmitted unidentified Enterobiinae may be explained by intensified social contacts between potential mates during the reproductive season starting in August/September in both mouse lemur species (Rina Evasoa et al., 2018).

#### 4.2.2 Host species-specific variation

The statistical analyses revealed no significant difference in parasite prevalence or egg shedding intensity for any helminth species between the two mouse lemur species. This result was unexpected, since sociality has been identified as an important risk factor for parasitic infections in a classical fitness trade-off associated with group-living (Patterson and Ruckstuhl, 2013). Observations on sleeping site behavior of a subset of radiocollared individuals of the study population confirmed the collective use of sleeping sites by *M. ravelobensis* and solitary sleeping habits of male *M. murinus* (Radespiel et al., 2003). Contrary to previous findings, female *M. murinus* were also found sleeping predominantly solitary (Klein et al., 2018). We therefore expected a difference in the likelihood of infection with homoxenous intestinal parasites between the two studied mouse lemur species as a result of the marked difference in sleeping site sociality. For example, comparisons of different primate hosts sharing the same habitat in western Madagascar revealed higher prevalences of directly transmitted intestinal parasites in group-living lemur species compared to solitary foragers (Springer and Kappeler, 2016). In contrast to our expectations, the more gregarious *M. ravelobensis* however, did not have a statistically significant higher likelihood of infection with directly transmitted Enterobiinae, *Lemuricola* or ascarid species.

Similar to the life cycle of well-studied Oxyuridae of humans, we assume oviposition in the perianal area and fecal-oral transmission for unidentified Enterobiinae and *Lemuricola* species. Ingestion of infective eggs during autogrooming maintains the parasitic cycle and close body contact and social grooming facilitate infection of conspecifics. The total prevalence for unidentified Enterobiinae was indeed higher for the group-sleeping *M. ravelobensis*, but this factor was not statistically significant in the multivariate statistical model. The frequent detection of unidientified Enterobiinae eggs in fecal samples, however, indicates that environmental contamination and indirect transmission in a shared habitat may also play a notable role alongside allo- and autogrooming.

#### 4.2.3 Sex-differences in intestinal parasite infection

We did not detect a sex bias towards males in any gastrointestinal helminth species nor in helminth species richness. Higher susceptibility to parasitic infections in adult males has been attributed to immunosuppressive properties of testosterone (Folstad and Karter, 1992). This suspected relationship was, for example, supported by a study on male chimpanzees where social rank and fecal testosterone levels were directly correlated with helminth parasite richness (Muehlenbein and Watts, 2010). However, hormonally mediated sex-specific differences in parasitism are often small and mostly observed under controlled experimental conditions (Schalk and Forbes, 1997). Additional determinants of a male-bias in parasitic infections could be variation in body mass and or behavior. *Microcebus* species are sexually monomorphic, but males increase their home range size during the period of reproductive activity (Ehresmann 2000).

Rather similar fecal testosterone levels in males and females have been reported for a congener, the brown mouse lemur *M. rufus* (Zohdy et al., 2014), which could, if verified for other species and across seasons, make the immunocompetence handicap hypothesis irrelevant for mouse lemurs. On the other hand, Hämäläinen et al. (2015a) did report a higher prevalence of infection with nematodes and cestodes in male *M. murinus* in the dry season, and discussed a potential relation to elevated sex hormones at the time of sampling. An annual variation in testis size and corresponding changes in testosterone levels have been shown in *M. murinus* in captivity (Petter-Rousseaux and Picon, 1981). The temporary increase in testis size during the reproductive season was however not associated statistically with higher helminth occurrence or egg shedding intensity in the present study.

#### 4.2.4 Age-dependent variation

Host age has previously been identified as a potential predictive variable for intestinal parasite infections with varying effects. While an accumulation of parasites with age may lead to higher infection levels in older individuals, susceptibility to infection may decrease with increasing age due to reinforced immunity following repeated contact to various parasites (MacIntosh et al., 2010). A study on *M. murinus* in western Madagascar found a decrease in intestinal parasite prevalence and richness with age (Hämäläinen et al., 2015b). However, the authors built their models on long-term longitudinal life history data of adult individuals with detailed capture histories, which varied between 1 and 10 years in age. In the present study, age classification at the moment of capture was only binary (juvenile/adult) and was based on a combination of body mass, body size and the presence/absence of large testes or estrous during the reproductive season. A characteristic peak in infection with host age has been previously described for several helminth species (Woolhouse, 1998), shaped by the encounter of infective parasite stages and the development of acquired immunity of the host. This may explain the significant influence of the host's developmental stage on the likelihood of *S. baeri* egg shedding, with more adults shedding eggs than juveniles in the present study. Our binary age classification limits statistical analysis and may therefore not reveal potential effects of acquired immunocompetence, but only represent accumulation of parasites from infancy to adulthood.

#### 4.2.5 Egg-shedding intensity

The informative value of EPGs needs to be treated cautiously, since egg output in fecal matter is influenced by various factors (e.g. host immunity, fecal consistency, parasite fecundity, size, age, and sex ratio) and a linear relationship between parasite burden and egg production has only been shown for few taxa (Stear et al., 1995). EPG counts, however, do provide information on the environmental contamination and infection pressure. Higher egg counts were observed in parallel with the seasonal increase in occurrence of *S. baeri*. Accumulation of parasite eggs in the environment increases the likelihood of infection for intermediate hosts, leading to higher infection rates of definitive hosts in a self-reinforcing process. This pattern was, however, only statistically detectable in *S. baeri* as the most common intestinal parasite species.

### 4.3 Intestinal helminths and mouse lemur body condition

Gastrointestinal parasites are often used as an indicator to assess the health status in wildlife populations, considering the potential nutrient extraction by endoparasitic organisms. The variation in body mass of adult mouse lemurs in the present study, however, was best explained by monthly and species-specific differences, whereas gastrointestinal helminth infections did not predict host body condition. However, the concurrent increase of helminth prevalence and decrease of food availability and body mass over the course of the dry season is indicative of a synergistic effect of nutritional status and parasitism on body mass. This interaction has previously been shown in livestock (Gulland, 1992) and is discussed in several primate species (Chapman et al., 2006; Harris et al., 2010; Agostini et al., 2017). A comprehensive analysis, including not only gastrointestinal parasites, but also ecto- and hemoparasites as well as climatic factors and phenological data reflecting food availability would be needed to study the multifactorial process of host-parasite-environment interactions.

## 5 Conclusion

Taken together, the combination of morphologic and molecular analysis of gastrointestinal parasite species in *M. murinus* and *M. ravelobensis* in the present study enables investigation of these helminth species across different host taxa. In the future, molecular studies are recommended that make use of high-throughput sequencing approaches that are gaining importance in the field of disease surveillance and conservation medicine. This may help to reveal potential transmission pathways not only within protected wildlife habitats, but also at the human-livestock-wildlife interface in future studies.

## Funding

The field research was supported by the Primate Conservation Inc. (Research Grant PCI# 1299), the German Academic Exchange Service (DAAD, travel grant no. 57212311) and the Gesellschaft für Primatologie (GfP, Christian-Vogel Fond).

## Ethics statement

All animal handling and sampling procedures complied with national and international animal welfare guidelines and were authorized by the Ministére de l’Environment, des Eaux et Forêts Malgache (Autorisation de recherche N° 063/15/MEEMEF/SG/DGF/DCB.SAP/SCB du March 12, 2015, Autorisation de recherche N° 34/16/MEEMEF/SG/DGF/DAPT/SCBT.Re [Renouvellement de l’Aut N° 210/15 du August 27, 2015]).

## CRediT authorship contribution statement

**Annette Klein:** Writing – original draft, Methodology, Investigation, Funding acquisition, Formal analysis. **Ute Radespiel:** Writing – review & editing, Supervision, Project administration, Conceptualization. **Andrea Springer:** Writing – review & editing, Formal analysis. **Romule Rakotondravony:** Writing – review & editing. **Christina Strube:** Writing – review & editing, Supervision, Project administration, Conceptualization.

## Declaration of competing interest

The authors declare that they have no known competing financial interests or personal relationships that could have appeared to influence the work reported in this paper.
