# Supplementary material for: Temporal dynamics in gastrointestinal helminth infections of sympatric mouse lemur species (Microcebus murinus and Microcebus ravelobensis) in Northwestern Madagascar
Source: Int J Parasitol Parasites Wildl. 2024 Aug 5;25:100972. doi: 10.1016/j.ijppaw.2024.100972 (PMC11369387; doi:10.1016/j.ijppaw.2024.100972)
Supplement: Multimedia component 1 [file mmc1.docx]

**Additional Table 1:** Individual mouse lemur fecal samples per month.

| Animal ID | Species | Sex | March 2016 | April 2015/16 | May 2015/16 | June 2015 | July 2015 | August 2015 | September 2015 | October 2015 | November 2015 | **Total** |
| --- | --- | --- | --- | --- | --- | --- | --- | --- | --- | --- | --- | --- |
| 01-15 | *M. ravelobensis* | female | 0 | 1 | 0 | 0 | 0 | 0 | 0 | 0 | 0 | **1** |
| 01-16 | *M. murinus* | female | 1 | 0 | 0 | 0 | 0 | 0 | 0 | 0 | 0 | **1** |
| 02-13 | *M. murinus* | female | 0 | 0 | 1 | 0 | 0 | 0 | 0 | 0 | 0 | **1** |
| 02-15 | *M. ravelobensis* | female | 0 | 1 | 0 | 0 | 0 | 0 | 0 | 0 | 0 | **1** |
| 02-16 | *M. murinus* | male | 1 | 2 | 0 | 0 | 0 | 0 | 0 | 0 | 0 | **3** |
| 03-15 | *M. murinus* | female | 0 | 1 | 0 | 2 | 2 | 2 | 2 | 2 | 1 | **12** |
| 03-16 | *M. murinus* | female | 1 | 0 | 0 | 0 | 0 | 0 | 0 | 0 | 0 | **1** |
| 04-15 | *M. ravelobensis* | male | 0 | 2 | 0 | 0 | 0 | 0 | 0 | 0 | 0 | **2** |
| 04-16 | *M. murinus* | female | 1 | 0 | 0 | 0 | 0 | 0 | 0 | 0 | 0 | **1** |
| 05-15 | *M. ravelobensis* | female | 0 | 2 | 1 | 0 | 0 | 0 | 0 | 0 | 0 | **3** |
| 05-16 | *M. murinus* | female | 1 | 1 | 2 | 0 | 0 | 0 | 0 | 0 | 0 | **4** |
| 06-15 | *M. murinus* | female | 0 | 1 | 1 | 2 | 0 | 2 | 2 | 2 | 1 | **11** |
| 06-16 | *M. murinus* | female | 0 | 1 | 0 | 0 | 0 | 0 | 0 | 0 | 0 | **1** |
| 07-15 | *M. murinus* | male | 0 | 1 | 3 | 2 | 2 | 1 | 1 | 2 | 1 | **13** |
| 07-16 | *M. murinus* | male | 0 | 1 | 1 | 0 | 0 | 0 | 0 | 0 | 0 | **2** |
| 08-15 | *M. ravelobensis* | male | 0 | 2 | 0 | 0 | 1 | 0 | 0 | 0 | 0 | **3** |
| 08-16 | *M. ravelobensis* | male | 0 | 1 | 0 | 0 | 0 | 0 | 0 | 0 | 0 | **1** |
| 09-15 | *M. murinus* | male | 0 | 1 | 2 | 0 | 0 | 0 | 0 | 0 | 0 | **3** |
| 09-16 | *M. ravelobensis* | female | 0 | 1 | 0 | 0 | 0 | 0 | 0 | 0 | 0 | **1** |
| 10-15 | *M. ravelobensis* | male | 0 | 1 | 4 | 0 | 0 | 0 | 0 | 0 | 0 | **5** |
| 10-16 | *M. ravelobensis* | male | 0 | 1 | 1 | 0 | 0 | 0 | 0 | 0 | 0 | **2** |
| 100-15 | *M. murinus* | female | 0 | 0 | 0 | 0 | 1 | 0 | 1 | 0 | 0 | **2** |
| 101-15 | *M. ravelobensis* | female | 0 | 0 | 0 | 0 | 1 | 2 | 1 | 2 | 1 | **7** |
| 102-15 | *M. murinus* | male | 1 | 0 | 0 | 0 | 1 | 0 | 2 | 2 | 1 | **7** |
| 103-15 | *M. ravelobensis* | male | 0 | 0 | 0 | 0 | 1 | 2 | 2 | 2 | 0 | **7** |
| 104-15 | *M. ravelobensis* | male | 0 | 0 | 0 | 0 | 1 | 1 | 0 | 1 | 0 | **3** |
| 105-15 | *M. ravelobensis* | male | 0 | 0 | 0 | 0 | 1 | 0 | 0 | 0 | 0 | **1** |
| 106-15 | *M. ravelobensis* | male | 0 | 2 | 2 | 0 | 1 | 2 | 2 | 2 | 1 | **12** |
| 107-15 | *M. ravelobensis* | male | 0 | 0 | 0 | 0 | 0 | 2 | 2 | 2 | 0 | **6** |
| 108-15 | *M. murinus* | male | 0 | 0 | 0 | 0 | 0 | 2 | 2 | 2 | 1 | **7** |
| 109-15 | *M. murinus* | male | 0 | 0 | 0 | 0 | 0 | 1 | 0 | 0 | 0 | **1** |
| 11-15 | *M. murinus* | male | 0 | 1 | 1 | 3 | 1 | 1 | 0 | 0 | 0 | **7** |
| 11-16 | *M. murinus* | female | 0 | 1 | 1 | 0 | 0 | 0 | 0 | 0 | 0 | **2** |
| 110-15 | *M. ravelobensis* | female | 0 | 0 | 0 | 0 | 0 | 1 | 0 | 0 | 0 | **1** |
| 111-15 | *M. ravelobensis* | female | 0 | 0 | 0 | 0 | 0 | 1 | 0 | 0 | 0 | **1** |
| 112-15 | *M. murinus* | male | 0 | 0 | 0 | 0 | 0 | 1 | 0 | 0 | 0 | **1** |
| 113-15 | *M. murinus* | male | 0 | 0 | 0 | 0 | 0 | 1 | 1 | 1 | 1 | **4** |
| 114-15 | *M. murinus* | male | 0 | 0 | 0 | 0 | 0 | 1 | 1 | 2 | 0 | **4** |
| 115-15 | *M. ravelobensis* | male | 2 | 2 | 2 | 0 | 0 | 0 | 3 | 2 | 1 | **12** |
| 116-15 | *M. ravelobensis* | male | 0 | 0 | 0 | 0 | 0 | 0 | 2 | 1 | 0 | **3** |
| 117-15 | *M. murinus* | female | 0 | 1 | 0 | 0 | 0 | 0 | 1 | 0 | 0 | **2** |
| 118-15 | *M. ravelobensis* | male | 0 | 0 | 0 | 0 | 0 | 0 | 2 | 1 | 0 | **3** |
| 119-15 | *M. murinus* | male | 0 | 0 | 0 | 0 | 0 | 0 | 1 | 0 | 0 | **1** |
| 12-15 | *M. murinus* | female | 0 | 2 | 2 | 0 | 1 | 0 | 2 | 2 | 0 | **9** |
| 12-16 | *M. murinus* | male | 0 | 1 | 0 | 0 | 0 | 0 | 0 | 0 | 0 | **1** |
| 120-15 | *M. murinus* | male | 0 | 0 | 0 | 0 | 0 | 0 | 1 | 0 | 0 | **1** |
| 121-15 | *M. ravelobensis* | male | 0 | 0 | 0 | 0 | 0 | 0 | 1 | 0 | 0 | **1** |
| 122-15 | *M. murinus* | male | 0 | 0 | 0 | 0 | 0 | 0 | 1 | 1 | 0 | **2** |
| 123-15 | *M. murinus* | male | 0 | 0 | 0 | 0 | 0 | 0 | 1 | 0 | 0 | **1** |
| 124-15 | *M. murinus* | female | 0 | 0 | 0 | 0 | 0 | 0 | 1 | 0 | 0 | **1** |
| 125-15 | *M. murinus* | Male | 0 | 0 | 0 | 0 | 0 | 0 | 1 | 1 | 0 | **2** |
| 126-15 | *M. murinus* | male | 0 | 0 | 0 | 0 | 0 | 0 | 1 | 0 | 0 | **1** |
| 127-15 | *M. ravelobensis* | female | 0 | 0 | 0 | 0 | 0 | 0 | 1 | 0 | 0 | **1** |
| 128-15 | *M. ravelobensis* | female | 0 | 0 | 0 | 0 | 0 | 0 | 0 | 1 | 0 | **1** |
| 129-15 | *M. ravelobensis* | male | 0 | 0 | 0 | 0 | 0 | 0 | 0 | 2 | 1 | **3** |
| 13-15 | *M. murinus* | male | 0 | 1 | 2 | 1 | 1 | 1 | 2 | 1 | 1 | **10** |
| 13-16 | *M. ravelobensis* | female | 0 | 1 | 2 | 0 | 0 | 0 | 0 | 0 | 0 | **3** |
| 130-15 | *M. murinus* | female | 0 | 1 | 0 | 0 | 0 | 0 | 0 | 1 | 0 | **2** |
| 131-15 | *M. ravelobensis* | female | 0 | 0 | 1 | 0 | 0 | 0 | 0 | 1 | 0 | **2** |
| 132-15 | *M. ravelobensis* | male | 0 | 0 | 0 | 0 | 0 | 0 | 0 | 1 | 1 | **2** |
| 133-15 | *M. ravelobensis* | male | 0 | 0 | 0 | 0 | 0 | 0 | 0 | 1 | 0 | **1** |
| 134-15 | *M. murinus* | female | 0 | 0 | 0 | 0 | 0 | 0 | 0 | 1 | 0 | **1** |
| 135-15 | *M. murinus* | male | 0 | 0 | 0 | 0 | 0 | 0 | 0 | 1 | 0 | **1** |
| 136-15 | *M. ravelobensis* | male | 0 | 0 | 0 | 0 | 0 | 0 | 0 | 0 | 1 | **1** |
| 14-15 | *M. ravelobensis* | male | 0 | 0 | 1 | 2 | 2 | 0 | 0 | 0 | 0 | **5** |
| 14-16 | *M. murinus* | female | 0 | 1 | 2 | 0 | 0 | 0 | 0 | 0 | 0 | **3** |
| 15-15 | *M. murinus* | male | 0 | 0 | 3 | 2 | 2 | 2 | 2 | 1 | 0 | **10** |
| 15-16 | *M. ravelobensis* | female | 0 | 1 | 1 | 0 | 0 | 0 | 0 | 0 | 0 | **2** |
| 16-15 | *M. murinus* | female | 0 | 0 | 1 | 0 | 0 | 0 | 0 | 0 | 0 | **1** |
| 16-16 | *M. murinus* | male | 0 | 1 | 1 | 0 | 0 | 0 | 0 | 0 | 0 | **2** |
| 17-15 | *M. ravelobensis* | female | 0 | 0 | 3 | 0 | 0 | 0 | 0 | 0 | 0 | **3** |
| 17-16 | *M. murinus* | male | 0 | 1 | 1 | 0 | 0 | 0 | 0 | 0 | 0 | **2** |
| 18-15 | *M. murinus* | female | 0 | 0 | 1 | 0 | 0 | 0 | 0 | 0 | 0 | **1** |
| 18-16 | *M. murinus* | male | 0 | 1 | 0 | 0 | 0 | 0 | 0 | 0 | 0 | **1** |
| 19-15 | *M. murinus* | female | 1 | 0 | 1 | 1 | 0 | 0 | 1 | 0 | 1 | **5** |
| 19-16 | *M. ravelobensis* | female | 0 | 0 | 1 | 0 | 0 | 0 | 0 | 0 | 0 | **1** |
| 20-15 | *M. ravelobensis* | female | 0 | 0 | 3 | 0 | 0 | 0 | 0 | 0 | 0 | **3** |
| 20-16 | *M. ravelobensis* | male | 0 | 0 | 1 | 0 | 0 | 0 | 0 | 0 | 0 | **1** |
| 21-15 | *M. murinus* | male | 0 | 0 | 2 | 2 | 0 | 0 | 0 | 0 | 0 | **4** |
| 21-16 | *M. ravelobensis* | male | 0 | 0 | 1 | 0 | 0 | 0 | 0 | 0 | 0 | **1** |
| 22-15 | *M. ravelobensis* | male | 0 | 0 | 2 | 0 | 0 | 0 | 0 | 0 | 0 | **2** |
| 22-16 | *M. ravelobensis* | female | 0 | 0 | 2 | 0 | 0 | 0 | 0 | 0 | 0 | **2** |
| 23-15 | *M. murinus* | female | 0 | 0 | 1 | 1 | 1 | 1 | 0 | 0 | 0 | **4** |
| 23-16 | *M. ravelobensis* | male | 0 | 0 | 2 | 0 | 0 | 0 | 0 | 0 | 0 | **2** |
| 24-15 | *M. ravelobensis* | male | 0 | 0 | 0 | 1 | 1 | 0 | 1 | 1 | 1 | **5** |
| 24-16 | *M. murinus* | female | 0 | 0 | 1 | 0 | 0 | 0 | 0 | 0 | 0 | **1** |
| 25-15 | *M. ravelobensis* | male | 0 | 0 | 2 | 0 | 0 | 0 | 0 | 0 | 0 | **2** |
| 25-16 | *M. murinus* | male | 0 | 0 | 1 | 0 | 0 | 0 | 0 | 0 | 0 | **1** |
| 26-14 | *M. ravelobensis* | female | 0 | 0 | 0 | 1 | 2 | 1 | 1 | 1 | 0 | **6** |
| 26-15 | *M. ravelobensis* | female | 0 | 0 | 1 | 0 | 0 | 0 | 0 | 0 | 0 | **1** |
| 26-16 | *M. ravelobensis* | male | 0 | 0 | 1 | 0 | 0 | 0 | 0 | 0 | 0 | **1** |
| 27-15 | *M. murinus* | female | 2 | 2 | 4 | 2 | 2 | 2 | 2 | 2 | 1 | **19** |
| 27-16 | *M. murinus* | female | 0 | 0 | 1 | 0 | 0 | 0 | 0 | 0 | 0 | **1** |
| 28-15 | *M. ravelobensis* | female | 0 | 0 | 2 | 0 | 0 | 0 | 0 | 0 | 0 | **2** |
| 28-16 | *M. murinus* | male | 0 | 0 | 1 | 0 | 0 | 0 | 0 | 0 | 0 | **1** |
| 29-15 | *M. murinus* | female | 0 | 0 | 2 | 3 | 2 | 1 | 2 | 1 | 0 | **11** |
| 30-15 | *M. murinus* | male | 0 | 0 | 3 | 0 | 0 | 0 | 0 | 0 | 0 | **3** |
| 30-16 | *M. murinus* | female | 0 | 0 | 1 | 0 | 0 | 0 | 0 | 0 | 0 | **1** |
| 31-15 | *M. ravelobensis* | female | 1 | 1 | 3 | 1 | 2 | 1 | 0 | 0 | 0 | **9** |
| 31-16 | *M. murinus* | male | 0 | 0 | 1 | 0 | 0 | 0 | 0 | 0 | 0 | **1** |
| 32-15 | *M. murinus* | male | 0 | 0 | 3 | 0 | 0 | 0 | 0 | 0 | 0 | **3** |
| 32-16 | *M. murinus* | male | 0 | 0 | 1 | 0 | 0 | 0 | 0 | 0 | 0 | **1** |
| 33-15 | *M. murinus* | female | 1 | 0 | 1 | 1 | 0 | 0 | 0 | 1 | 0 | **4** |
| 33-16 | *M. ravelobensis* | female | 0 | 0 | 1 | 0 | 0 | 0 | 0 | 0 | 0 | **1** |
| 34-15 | *M. ravelobensis* | male | 0 | 0 | 1 | 2 | 2 | 1 | 0 | 0 | 0 | **6** |
| 34-16 | *M. ravelobensis* | male | 0 | 0 | 1 | 0 | 0 | 0 | 0 | 0 | 0 | **1** |
| 35-15 | *M. ravelobensis* | female | 0 | 0 | 3 | 1 | 1 | 0 | 1 | 0 | 0 | **6** |
| 35-16 | *M. ravelobensis* | female | 0 | 0 | 1 | 0 | 0 | 0 | 0 | 0 | 0 | **1** |
| 36-15 | *M. ravelobensis* | female | 0 | 0 | 1 | 1 | 1 | 2 | 2 | 2 | 0 | **9** |
| 37-15 | *M. ravelobensis* | female | 0 | 0 | 2 | 0 | 0 | 0 | 0 | 0 | 0 | **2** |
| 38-15 | *M. ravelobensis* | female | 0 | 0 | 1 | 1 | 3 | 2 | 2 | 0 | 0 | **9** |
| 39-15 | *M. murinus* | female | 0 | 0 | 1 | 2 | 1 | 2 | 2 | 2 | 1 | **11** |
| 40-15 | *M. ravelobensis* | female | 1 | 2 | 4 | 3 | 1 | 0 | 0 | 0 | 0 | **11** |
| 41-10 | *M. murinus* | male | 0 | 1 | 3 | 1 | 2 | 2 | 0 | 0 | 0 | **9** |
| 41-11 | *M. murinus* | female | 0 | 2 | 3 | 2 | 1 | 0 | 0 | 0 | 0 | **8** |
| 41-15 | *M. ravelobensis* | female | 1 | 0 | 1 | 2 | 0 | 1 | 0 | 0 | 0 | **5** |
| 42-15 | *M. ravelobensis* | female | 0 | 2 | 2 | 2 | 2 | 1 | 2 | 2 | 1 | **14** |
| 43-15 | *M. murinus* | male | 0 | 0 | 2 | 0 | 1 | 2 | 3 | 1 | 0 | **9** |
| 44-15 | *M. ravelobensis* | female | 0 | 0 | 1 | 0 | 1 | 0 | 0 | 0 | 0 | **2** |
| 45-15 | *M. ravelobensis* | male | 0 | 0 | 1 | 0 | 0 | 0 | 0 | 0 | 0 | **1** |
| 46-15 | *M. ravelobensis* | male | 0 | 1 | 3 | 0 | 1 | 2 | 2 | 2 | 1 | **12** |
| 47-15 | *M. murinus* | male | 0 | 0 | 1 | 0 | 3 | 1 | 2 | 2 | 1 | **10** |
| 48-15 | *M. ravelobensis* | male | 0 | 0 | 1 | 2 | 1 | 2 | 2 | 2 | 1 | **11** |
| 49-15 | *M. ravelobensis* | female | 0 | 1 | 4 | 2 | 1 | 0 | 0 | 1 | 0 | **9** |
| 50-13 | *M. murinus* | male | 0 | 0 | 0 | 0 | 0 | 1 | 2 | 2 | 0 | **5** |
| 50-15 | *M. ravelobensis* | female | 0 | 0 | 3 | 1 | 1 | 1 | 3 | 1 | 1 | **11** |
| 51-15 | *M. ravelobensis* | male | 2 | 2 | 3 | 1 | 2 | 2 | 1 | 1 | 1 | **15** |
| 52-15 | *M. ravelobensis* | male | 0 | 0 | 1 | 0 | 1 | 0 | 0 | 0 | 0 | **2** |
| 53-15 | *M. murinus* | male | 0 | 0 | 1 | 1 | 0 | 0 | 0 | 0 | 0 | **2** |
| 54-15 | *M. ravelobensis* | female | 2 | 2 | 3 | 2 | 2 | 2 | 2 | 2 | 1 | **18** |
| 55-15 | *M. ravelobensis* | female | 0 | 0 | 1 | 0 | 2 | 0 | 0 | 0 | 0 | **3** |
| 56-15 | *M. murinus* | female | 1 | 1 | 2 | 2 | 3 | 2 | 3 | 2 | 1 | **17** |
| 57-13 | *M. murinus* | female | 0 | 1 | 2 | 1 | 0 | 1 | 1 | 0 | 0 | **6** |
| 57-15 | *M. ravelobensis* | female | 0 | 0 | 1 | 1 | 0 | 0 | 0 | 0 | 0 | **2** |
| 58-15 | *M. murinus* | female | 0 | 2 | 2 | 1 | 0 | 1 | 2 | 0 | 1 | **9** |
| 59-15 | *M. ravelobensis* | female | 0 | 0 | 0 | 2 | 1 | 0 | 0 | 0 | 0 | **3** |
| 60-15 | *M. ravelobensis* | male | 0 | 0 | 0 | 1 | 0 | 0 | 0 | 0 | 0 | **1** |
| 61-15 | *M. ravelobensis* | male | 0 | 0 | 0 | 2 | 3 | 2 | 1 | 1 | 0 | **9** |
| 62-15 | *M. murinus* | female | 0 | 0 | 0 | 1 | 0 | 0 | 0 | 0 | 0 | **1** |
| 63-15 | *M. ravelobensis* | male | 0 | 0 | 0 | 1 | 0 | 0 | 0 | 1 | 0 | **2** |
| 64-15 | *M. ravelobensis* | male | 0 | 1 | 0 | 2 | 2 | 3 | 2 | 2 | 1 | **13** |
| 65-15 | *M. ravelobensis* | female | 1 | 0 | 1 | 2 | 1 | 2 | 2 | 0 | 0 | **9** |
| 66-15 | *M. murinus* | male | 0 | 0 | 0 | 1 | 0 | 0 | 0 | 0 | 0 | **1** |
| 67-15 | *M. ravelobensis* | female | 1 | 1 | 1 | 1 | 3 | 2 | 2 | 1 | 1 | **13** |
| 68-15 | *M. ravelobensis* | female | 0 | 0 | 0 | 1 | 0 | 0 | 0 | 0 | 0 | **1** |
| 69-15 | *M. murinus* | male | 0 | 0 | 0 | 1 | 0 | 0 | 0 | 1 | 0 | **2** |
| 70-11 | *M. murinus* | male | 0 | 0 | 0 | 0 | 1 | 2 | 2 | 1 | 0 | **6** |
| 70-15 | *M. murinus* | female | 2 | 1 | 0 | 2 | 1 | 2 | 2 | 2 | 1 | **13** |
| 71-15 | *M. ravelobensis* | male | 0 | 0 | 0 | 2 | 2 | 0 | 1 | 1 | 0 | **6** |
| 72-15 | *M. ravelobensis* | female | 0 | 1 | 1 | 1 | 1 | 0 | 1 | 2 | 1 | **8** |
| 73-15 | *M. murinus* | female | 2 | 1 | 2 | 1 | 1 | 0 | 2 | 2 | 1 | **12** |
| 74-15 | *M. ravelobensis* | female | 0 | 0 | 0 | 1 | 0 | 0 | 0 | 0 | 0 | **1** |
| 75-15 | *M. ravelobensis* | male | 0 | 0 | 0 | 2 | 2 | 2 | 2 | 3 | 1 | **12** |
| 76-15 | *M. ravelobensis* | female | 0 | 1 | 1 | 1 | 2 | 0 | 0 | 0 | 0 | **5** |
| 77-15 | *M. ravelobensis* | female | 0 | 0 | 0 | 1 | 2 | 0 | 1 | 0 | 0 | **4** |
| 78-15 | *M. ravelobensis* | female | 1 | 1 | 2 | 2 | 2 | 1 | 0 | 0 | 0 | **9** |
| 79-15 | *M. ravelobensis* | female | 0 | 0 | 0 | 2 | 0 | 0 | 0 | 0 | 0 | **2** |
| 80-15 | *M. murinus* | female | 0 | 0 | 0 | 1 | 1 | 0 | 0 | 0 | 0 | **2** |
| 81-15 | *M. ravelobensis* | female | 0 | 0 | 0 | 1 | 2 | 2 | 2 | 2 | 1 | **10** |
| 82-15 | *M. ravelobensis* | male | 0 | 0 | 0 | 1 | 0 | 2 | 2 | 2 | 1 | **8** |
| 83-15 | *M. ravelobensis* | female | 0 | 0 | 0 | 1 | 1 | 0 | 0 | 0 | 0 | **2** |
| 84-15 | *M. ravelobensis* | female | 0 | 0 | 0 | 1 | 0 | 0 | 0 | 0 | 0 | **1** |
| 85-15 | *M. ravelobensis* | male | 0 | 0 | 0 | 1 | 0 | 0 | 0 | 0 | 0 | **1** |
| 86-15 | *M. ravelobensis* | female | 0 | 0 | 0 | 1 | 1 | 0 | 0 | 0 | 0 | **2** |
| 87-15 | *M. ravelobensis* | male | 0 | 0 | 0 | 1 | 2 | 0 | 2 | 0 | 1 | **6** |
| 88-15 | *M. murinus* | female | 0 | 0 | 0 | 0 | 1 | 0 | 0 | 0 | 0 | **1** |
| 89-15 | *M. ravelobensis* | female | 0 | 0 | 0 | 0 | 2 | 2 | 2 | 2 | 1 | **9** |
| 90-15 | *M. ravelobensis* | male | 0 | 0 | 1 | 0 | 1 | 0 | 0 | 0 | 0 | **2** |
| 91-15 | *M. murinus* | male | 0 | 0 | 0 | 0 | 2 | 0 | 0 | 1 | 0 | **3** |
| 92-15 | *M. ravelobensis* | male | 0 | 0 | 0 | 0 | 1 | 1 | 0 | 1 | 1 | **4** |
| 93-15 | *M. ravelobensis* | female | 0 | 0 | 0 | 0 | 2 | 1 | 0 | 1 | 0 | **4** |
| 94-15 | *M. murinus* | male | 0 | 1 | 0 | 0 | 2 | 2 | 2 | 2 | 1 | **10** |
| 95-15 | *M. murinus* | female | 0 | 0 | 0 | 0 | 1 | 0 | 0 | 0 | 0 | **1** |
| 96-15 | *M. ravelobensis* | female | 2 | 2 | 2 | 0 | 2 | 2 | 2 | 1 | 1 | **14** |
| 97-15 | *M. ravelobensis* | female | 0 | 0 | 0 | 0 | 0 | 2 | 2 | 2 | 1 | **7** |
| 98-15 | *M. ravelobensis* | female | 0 | 0 | 0 | 0 | 1 | 0 | 0 | 0 | 0 | **1** |
| 99-15 | *M. ravelobensis* | male | 0 | 0 | 0 | 0 | 1 | 1 | 2 | 1 | 0 | **5** |
