# Supplementary material for: Temporal dynamics in gastrointestinal helminth infections of sympatric mouse lemur species (Microcebus murinus and Microcebus ravelobensis) in Northwestern Madagascar
Source: Int J Parasitol Parasites Wildl. 2024 Aug 5;25:100972. doi: 10.1016/j.ijppaw.2024.100972 (PMC11369387; doi:10.1016/j.ijppaw.2024.100972)
Supplement: Multimedia component 2 [file mmc2.docx]

**Additional Table 2:** Results of generalized linear mixed models (GLMM, logit link, binomal assumption), and subsequent pairwise comparisons between months, respectively, for *S. baeri*, unidentified Enterobiinae and *Spirura* sp.

| **Factor** | **Estimate** | **SE** | ***P*-value** | **Effect on parasite** |
| --- | --- | --- | --- | --- |
| ***Subulura baeri***  Best model: sampling month + age (adult, juvenile) + coinfection with unidentified Enterobiinae + coinfection with *Spirura* sp. + coinfection with *Lemuricola* sp. + coinfection with *Hymenolepis* sp.1  Null model comparison: Deviance = 913.46, Df = 13, *P* < 0.001*** | | | | |
| Month |  |  |  |  |
| April *vs* March | 0.795 | 0.521 | 0.836 |  |
| May *vs* March | 0.282 | 0.486 | 1.000 |  |
| June *vs* March | 0.855 | 0.509 | 0.747 |  |
| July *vs* March | 1.121 | 0.501 | 0.368 |  |
| August *vs* March | 2.010 | 0.524 | 0.004** | August > March |
| September *vs* March | 2.796 | 0.545 | < 0.001*** | September > March |
| October *vs* March | 1.985 | 0.517 | 0.004** | October > March |
| November *vs* March | 1.183 | 0.570 | 0.476 |  |
| May *vs* April | -0.513 | 0.323 | 0.802 |  |
| June *vs* April | 0.060 | 0.349 | 1.000 |  |
| July *vs* April | 0.327 | 0.340 | 0.988 |  |
| August *vs* April | 1.216 | 0.370 | 0.027* | August > April |
| September *vs* April | 2.002 | 0.400 | < 0.001*** | September > April |
| October *vs* April | 1.191 | 0.366 | 0.030* | October > April |
| November *vs* April | 0.389 | 0.440 | 0.993 |  |
| June *vs* May | 0.573 | 0.293 | 0.560 |  |
| July *vs* May | 0.839 | 0.281 | 0.067 |  |
| August *vs* May | 1.728 | 0.319 | < 0.001*** | August > May |
| September *vs* May | 2.514 | 0.353 | < 0.001*** | September > May |
| October *vs* May | 1.703 | 0.317 | < 0.001*** | October > May |
| November *vs* May | 0.901 | 0.402 | 0.364 |  |
| July *vs* June | 0.266 | 0.301 | 0.993 |  |
| August *vs* June | 1.155 | 0.334 | 0.015* | August > June |
| September *vs* June | 1.941 | 0.369 | < 0.001*** | September > June |
| October *vs* June | 1.130 | 0.340 | 0.023* | October > June |
| November *vs* June | 0.328 | 0.424 | 0.997 |  |
| August *vs* July | 0.889 | 0.322 | 0.119 |  |
| September *vs* July | 1.675 | 0.357 | < 0.001*** | September > July |
| October *vs* July | 0.864 | 0.328 | 0.163 |  |
| November *vs* July | 0.062 | 0.415 | 1.000 |  |
| September *vs* August | 0.786 | 0.375 | 0.463 |  |
| October *vs* August | -0.025 | 0.351 | 1.000 |  |
| November *vs* August | -0.827 | 0.435 | 0.600 |  |
| October *vs* September | -0.811 | 0.380 | 0.436 |  |
| November *vs* September | -1.613 | 0.458 | 0.012* | November < September |
| November *vs* October | -0.802 | 0.429 | 0.620 |  |
| Age (ref. juvenile) | -0.878 | 0.237 | < 0.001*** | Adult > juvenile |
| Coinfection with unidentified Enterobiinae (ref. positive) | 0.884 | 0.237 | < 0.001*** | Higher likelihood of infection when positive for unidentified Enterobiinae |
| Coinfection with *Spirura* sp. (ref. positive) | 1.385 | 5.190 | < 0.001*** | Higher likelihood of infection when positive for *Spirura* sp. |
| Coinfection with *Lemuricola* sp. (ref. positive) | -1.845 | 0.752 | 0.014* | Lower likelihood of infection when positive for *Lemuricola* sp. |
| Coinfection with *Hymenolepis* sp. 1 (ref. positive) | 1.234 | 0.649 | 0.0573 |  |
| **Unidentified Enterobiinae**  Best model: sampling month + coinfection with *S. baeri*  Null model comparison: Deviance = 709.39, Df = 9, *P* < 0.001*** | | | | |
| Month |  |  |  |  |
| April *vs* March | 15.910 | 45.797 | 1.000 |  |
| May *vs* March | 16.338 | 45.795 | 1.000 |  |
| June *vs* March | 16.780 | 45.795 | 1.000 |  |
| July *vs* March | 17.273 | 45.795 | 1.000 |  |
| August *vs* March | 16.907 | 45.795 | 1.000 |  |
| September *vs* March | 17.427 | 45.795 | 1.000 |  |
| October *vs* March | 16.441 | 45.796 | 1.000 |  |
| November *vs* March | 14.468 | 45.806 | 1.000 |  |
| May *vs* April | 0.428 | 0.502 | 0.993 |  |
| June *vs* April | 0.870 | 0.513 | 0.694 |  |
| July *vs* April | 1.362 | 0.484 | 0.085 |  |
| August *vs* April | 0.997 | 0.503 | 0.490 |  |
| September *vs* April | 1.517 | 0.481 | 0.031* | September > April |
| October *vs* April | 0.531 | 0.512 | 0.975 |  |
| November *vs* April | -1.443 | 1.104 | 0.906 |  |
| June *vs* May | 0.441 | 0.373 | 0.946 |  |
| July *vs* May | 0.934 | 0.335 | 0.091 |  |
| August *vs* May | 0.569 | 0.366 | 0.785 |  |
| September *vs* May | 1.089 | 0.336 | 0.024* | September > May |
| October *vs* May | 0.103 | 0.380 | 1.000 |  |
| November *vs* May | -1.870 | 1.050 | 0.634 |  |
| July *vs* June | 0.493 | 0.348 | 0.860 |  |
| August *vs* June | 0.127 | 0.377 | 1.000 |  |
| September *vs* June | 0.647 | 0.346 | 0.569 |  |
| October *vs* June | -0.339 | 0.390 | 0.992 |  |
| November *vs* June | -2.312 | 1.054 | 0.348 |  |
| August *vs* July | -0.365 | 0.336 | 0.966 |  |
| September *vs* July | 0.155 | 0.300 | 1.000 |  |
| October *vs* July | -0.832 | 0.350 | 0.244 |  |
| November *vs* July | -2.805 | 1.040 | 0.115 |  |
| September *vs* August | 0.520 | 0.323 | 0.750 |  |
| October *vs* August | -0.466 | 0.370 | 0.923 |  |
| November *vs* August | -2.439 | 1.047 | 0.267 |  |
| October *vs* September | -0.986 | 0.335 | 0.059 |  |
| November *vs* September | -2.959 | 1.037 | 0.076 |  |
| November *vs* October | -1.973 | 1.051 | 0.565 |  |
| Coinfection with *S. baeri* (ref. positive) | 0.840 | 0.222 | < 0.001*** | Higher likelihood of infection when positive for *S. baeri* |
| ***Spirura* sp.**  Best model: sampling month + coinfection with *S. baeri*  Null model comparison: Deviance = 612.22, Df = 9, *P* < 0.001*** | | | | |
| Month |  |  |  |  |
| April *vs* March | -0.395 | 0.687 | 1.000 |  |
| May *vs* March | -1.046 | 0.660 | 0.799 |  |
| June *vs* March | -2.898 | 0.954 | 0.055 |  |
| July *vs* March | -1.763 | 0.720 | 0.241 |  |
| August *vs* March | -1.093 | 0.686 | 0.793 |  |
| September *vs* March | 0.289 | 0.642 | 1.000 |  |
| October *vs* March | 0.402 | 0.641 | 0.999 |  |
| November *vs* March | 0.710 | 0.707 | 0.984 |  |
| May *vs* April | -0.651 | 0.503 | 0.927 |  |
| June *vs* April | -2.503 | 0.852 | 0.073 |  |
| July *vs* April | -1.368 | 0.575 | 0.277 |  |
| August *vs* April | -0.699 | 0.534 | 0.922 |  |
| September *vs* April | 0.683 | 0.479 | 0.877 |  |
| October *vs* April | 0.797 | 0.480 | 0.753 |  |
| November *vs* April | 1.104 | 0.571 | 0.568 |  |
| June *vs* May | -1.852 | 0.820 | 0.348 |  |
| July *vs* May | -0.717 | 0.524 | 0.901 |  |
| August *vs* May | -0.047 | 0.484 | 1.000 |  |
| September *vs* May | 1.335 | 0.425 | 0.040* | September > May |
| October *vs* May | 1.448 | 0.428 | 0.018* | October > May |
| November *vs* May | 1.756 | 0.531 | 0.024* | November > May |
| July *vs* June | 1.135 | 0.845 | 0.910 |  |
| August *vs* June | 1.804 | 0.826 | 0.394 |  |
| September *vs* June | 3.186 | 0.800 | 0.002** | September > June |
| October *vs* June | 3.300 | 0.803 | 0.001** | October > June |
| November *vs* June | 3.607 | 0.867 | 0.001** | November > June |
| August *vs* July | 0.669 | 0.519 | 0.928 |  |
| September *vs* July | 2.051 | 0.479 | < 0.001*** | September > July |
| October *vs* July | 2.164 | 0.487 | < 0.001*** | October > July |
| November *vs* July | 2.472 | 0.588 | < 0.001*** | November > July |
| September *vs* August | 1.382 | 0.416 | 0.023* | September > August |
| October *vs* August | 1.495 | 0.426 | 0.012* | October > August |
| November *vs* August | 1.803 | 0.540 | 0.022* | November > August |
| October *vs* September | 0.113 | 0.346 | 1.000 |  |
| November *vs* September | 0.421 | 0.4778 | 0.993 |  |
| November *vs* October | 0.308 | 0.477 | 0.999 |  |
| Coinfection with *S. baeri* (ref. positive) | 1.537 | 0.295 | < 0.001*** | Higher likelihood of infection when positive for *S. baeri* |
