# Supplementary material for: Temporal dynamics in gastrointestinal helminth infections of sympatric mouse lemur species (Microcebus murinus and Microcebus ravelobensis) in Northwestern Madagascar
Source: Int J Parasitol Parasites Wildl. 2024 Aug 5;25:100972. doi: 10.1016/j.ijppaw.2024.100972 (PMC11369387; doi:10.1016/j.ijppaw.2024.100972)
Supplement: Multimedia component 3 [file mmc3.docx]

**Additional Table 3:** Results of a linear mixed effects model (LME) investigating potential factors influencing egg shedding intensity (eggs per gram feces [EPG]), and subsequent pairwise comparisons between months for fecal samples positive for *S. baeri*.

| Factor | Estimate | SE | *P*-value | Effect on parasite |
| --- | --- | --- | --- | --- |
| Best model: sampling month (AIC = 767.634), EPG counts were log transformed  Null model comparison: likelihood ratio = 38.776, *P* < 0.001*** | | | | |
| Month |  |  |  |  |
| April *vs* March | 0.120 | 0.208 | 1.000 |  |
| May *vs* March | -0.055 | 0.200 | 1.000 |  |
| June *vs* March | -0.118 | 0.204 | 1.000 |  |
| July *vs* March | -0.011 | 0.198 | 1.000 |  |
| August *vs* March | 0.287 | 0.197 | 0.864 |  |
| September *vs* March | 0.484 | 0.192 | 0.207 |  |
| October *vs* March | 0.307 | 0.195 | 0.805 |  |
| November *vs* March | 0.138 | 0.216 | 0.999 |  |
| May *vs* April | -0.175 | 0.128 | 0.901 |  |
| June *vs* April | -0.238 | 0.134 | 0.677 |  |
| July *vs* April | -0.131 | 0.125 | 0.979 |  |
| August *vs* April | 0.167 | 0.123 | 0.904 |  |
| September *vs* April | 0.364 | 0.115 | 0.037* | September > April |
| October *vs* April | 0.187 | 0.119 | 0.807 |  |
| November *vs* April | 0.018 | 0.152 | 1.000 |  |
| June *vs* May | -0.063 | 0.118 | 1.000 |  |
| July *vs* May | 0.044 | 0.107 | 1.000 |  |
| August *vs* May | 0.342 | 0.106 | 0.030* | August > May |
| September *vs* May | 0.539 | 0.096 | < 0.001*** | September > May |
| October *vs* May | 0.362 | 0.101 | 0.009** | October > May |
| November *vs* May | 0.193 | 0.139 | 0.894 |  |
| July *vs* June | 0.107 | 0.114 | 0.990 |  |
| August *vs* June | 0.405 | 0.113 | 0.009** | August > June |
| September *vs* June | 0.602 | 0.104 | < 0.001*** | September > June |
| October *vs* June | 0.425 | 0.109 | 0.003** | October > June |
| November *vs* June | 0.256 | 0.144 | 0.680 |  |
| August *vs* July | 0.298 | 0.101 | 0.071 |  |
| September *vs* July | 0.495 | 0.091 | < 0.001*** | September > July |
| October *vs* July | 0.318 | 0.096 | 0.025* | October > July |
| November *vs* July | 0.149 | 0.135 | 0.971 |  |
| September *vs* August | 0.197 | 0.089 | 0.375 |  |
| October *vs* August | 0.020 | 0.094 | 1.000 |  |
| November *vs* August | -0.149 | 0.134 | 0.968 |  |
| October *vs* September | -0.177 | 0.083 | 0.433 |  |
| November *vs* September | -0.346 | 0.126 | 0.123 |  |
| November *vs* October | -0.169 | 0.130 | 0.925 |  |
