# Supplementary material for: Temporal dynamics in gastrointestinal helminth infections of sympatric mouse lemur species (Microcebus murinus and Microcebus ravelobensis) in Northwestern Madagascar
Source: Int J Parasitol Parasites Wildl. 2024 Aug 5;25:100972. doi: 10.1016/j.ijppaw.2024.100972 (PMC11369387; doi:10.1016/j.ijppaw.2024.100972)
Supplement: Multimedia component 4 [file mmc4.docx]

**Additional Table 4:** Results of linear mixed effects models (LMEs) for a potential influence of intestinal helminth occurrence on adult host body mass in addition to the fixed factors species, sex and month with subsequent pairwise comparisons between months (N = 154).

| **Factor** | **Estimate** | **SE** | ***P*-value** | **Effect on body mass** |
| --- | --- | --- | --- | --- |
| Best model: species + sex + month + *Spirura* sp. (presence/absence) (AIC = 3630.7)  Null model comparison: likelihood ratio = 247.430, *P* < 0.001*** | | | | |
| Species (ref. *M. ravelobensis*) | 3.013 | 1.081 | 0.006 * | *M. ravelobensis* > *M. murinus* |
| Sex (ref. male) | -1.855 | 1.066 | 0.084 |  |
| Month |  |  |  |  |
| April *vs* March | -0.447 | 0.906 | 1.000 |  |
| May *vs* March | -2.145 | 0.834 | 0.191 |  |
| June *vs* March | -6.636 | 0.886 | < 0.001*** | June < March |
| July *vs* March | -9.356 | 0.868 | < 0.001*** | July < March |
| August *vs* March | -7.279 | 0.904 | < 0.001*** | August < March |
| September *vs* March | -6.444 | 0.872 | < 0.001*** | September < March |
| October *vs* March | -7.077 | 0.865 | < 0.001*** | October < March |
| November *vs* March | -6.381 | 0.964 | < 0.001*** | November < March |
| May *vs* April | -1.698 | 0.662 | 0.193 |  |
| June *vs* April | -6.189 | 0.749 | < 0.001*** | June < April |
| July *vs* April | -8.909 | 0.720 | < 0.001*** | July < April |
| August *vs* April | -6.832 | 0.767 | < 0.001*** | August < April |
| September *vs* April | -5.997 | 0.725 | < 0.001*** | September < April |
| October *vs* April | -6.630 | 0.718 | < 0.001*** | October < April |
| November *vs* April | -5.934 | 0.835 | < 0.001*** | November < April |
| June *vs* May | -4.491 | 0.577 | < 0.001*** | June < May |
| July *vs* May | -7.212 | 0.550 | < 0.001*** | July < May |
| August *vs* May | -5.135 | 0.618 | < 0.001*** | August < May |
| September *vs* May | -4.299 | 0.569 | < 0.001*** | September < May |
| October *vs* May | -4.932 | 0.574 | < 0.001*** | October < May |
| November *vs* May | -4.236 | 0.721 | < 0.001*** | November < May |
| July *vs* June | -2.721 | 0.574 | < 0.001*** | July < June |
| August *vs* June | -0.644 | 0.643 | 0.985 |  |
| September *vs* June | 0.192 | 0.612 | 1.000 |  |
| October *vs* June | -0.441 | 0.616 | 0.998 |  |
| November *vs* June | 0.255 | 0.763 | 1.000 |  |
| August *vs* July | 2.077 | 0.602 | 0.015* | August > July |
| September *vs* July | 2.912 | 0.568 | < 0.001*** | September > July |
| October *vs* July | 2.279 | 0.577 | 0.003** | October > July |
| November *vs* July | 2.975 | 0.733 | 0.001** | November > July |
| September *vs* August | 0.835 | 0.601 | 0.897 |  |
| October *vs* August | 0.202 | 0.611 | 1.000 |  |
| November *vs* August | 0.898 | 0.765 | 0.960 |  |
| October *vs* September | -0.633 | 0.539 | 0.959 |  |
| November *vs* September | 0.063 | 0.712 | 1.000 |  |
| November *vs* October | 0.696 | 0.691 | 0.984 |  |
| *Spirura* sp. (ref. presence) | -0.627 | 0.449 | 0.163 |  |
| *Subulura baeri*  Model: species + sex + month + *Subulura baeri* (presence/absence) (AIC = 3631.5) | | | | |
| *S. baeri* (ref. presence) | 0.449 | 0.334 | 0.180 |  |
| Unidentified Enterobiinae  Model: species + sex + month + unidentified Enterobiinae (presence/absence) (AIC = 3631.8) | | | | |
| Unidentified Enterobiinae  (ref. presence) | 0.417 | 0.397 | 0.293 |  |
